# A Metagenomics-Based Metabolic Model of Nitrate-Dependent Anaerobic Oxidation of Methane by *Methanoperedens*-Like Archaea

**DOI:** 10.3389/fmicb.2015.01423

**Published:** 2015-12-18

**Authors:** Arslan Arshad, Daan R. Speth, Rob M. de Graaf, Huub J. M. Op den Camp, Mike S. M. Jetten, Cornelia U. Welte

**Affiliations:** Department of Microbiology, Institute for Water and Wetland Research, Radboud UniversityNijmegen, Netherlands

**Keywords:** methanotrophy, anaerobic respiration, archaea, methanogenesis, ANME, cytochrome *c*, heterodisulfide reductase, electron transport

## Abstract

Methane oxidation is an important process to mitigate the emission of the greenhouse gas methane and further exacerbating of climate forcing. Both aerobic and anaerobic microorganisms have been reported to catalyze methane oxidation with only a few possible electron acceptors. Recently, new microorganisms were identified that could couple the oxidation of methane to nitrate or nitrite reduction. Here we investigated such an enrichment culture at the (meta) genomic level to establish a metabolic model of nitrate-driven anaerobic oxidation of methane (nitrate-AOM). Nitrate-AOM is catalyzed by an archaeon closely related to (reverse) methanogens that belongs to the ANME-2d clade, tentatively named *Methanoperedens nitroreducens*. Methane may be activated by methyl-CoM reductase and subsequently undergo full oxidation to carbon dioxide via reverse methanogenesis. All enzymes of this pathway were present and expressed in the investigated culture. The genome of the archaeal enrichment culture encoded a variety of enzymes involved in an electron transport chain similar to those found in *Methanosarcina* species with additional features not previously found in methane-converting archaea. Nitrate reduction to nitrite seems to be located in the pseudoperiplasm and may be catalyzed by an unusual Nar-like protein complex. A small part of the resulting nitrite is reduced to ammonium which may be catalyzed by a Nrf-type nitrite reductase. One of the key questions is how electrons from cytoplasmically located reverse methanogenesis reach the nitrate reductase in the pseudoperiplasm. Electron transport in *M. nitroreducens* probably involves cofactor F_420_ in the cytoplasm, quinones in the cytoplasmic membrane and cytochrome *c* in the pseudoperiplasm. The membrane-bound electron transport chain includes F_420_H_2_ dehydrogenase and an unusual Rieske/cytochrome *b* complex. Based on genome and transcriptome studies a tentative model of how central energy metabolism of nitrate-AOM could work is presented and discussed.

## Introduction

Methane is an important greenhouse gas that is produced by microbiological processes, mainly methanogenesis in freshwater and marine ecosystems (Thauer et al., 2008) and the demethylation of methylphosphonates in the ocean (Metcalf et al., 2012). Part of the produced methane is oxidized by methanotrophic microorganisms leading to a reduced amount of methane escaping into the atmosphere. Methanotrophic microorganisms can be divided into two classes. Aerobic methanotrophs are bacteria that make use of the enzyme methane monooxygenase to activate the inert methane molecule (Sirajuddin and Rosenzweig, 2015). Anaerobic methanotrophs use an external electron acceptor other than oxygen and can be found in both prokaryotic domains, *Bacteria* and *Archaea* (Boetius et al., 2000; Ettwig et al., 2010; Haroon et al., 2013). Nitrite-dependent anaerobic oxidation of methane (AOM) is catalyzed by the anaerobic bacterium *Methylomirabilis oxyfera* that belongs to the NC10 phylum (Ettwig et al., 2010). After reduction of nitrite to NO it presumably dismutates NO to N_2_ and O_2_ to subsequently make use of the produced oxygen for an aerobic-type methane activation reaction via methane monooxygenase (Ettwig et al., 2010, 2012). All other anaerobic methanotrophs have been reported to belong to the domain *Archaea* and presumably use the reverse reaction of methyl-coenzyme M reductase—the key enzyme in methanogenesis—for the activation of methane (Krüger et al., 2003; Scheller et al., 2010). So far, enrichment cultures were reported to couple the oxidation of methane to the reduction of sulfate or nitrate (Boetius et al., 2000; Raghoebarsing et al., 2006; Haroon et al., 2013). Sulfate-dependent AOM seems to be catalyzed by the symbiotic association of an anaerobic methanotrophic archaeon (ANME) with a bacterial sulfate reducing partner (Knittel and Boetius, 2009; Ruff et al., 2015). Nitrate-dependent AOM, in contrast, seems to be catalyzed by an archaeal methanotroph alone that was named *Methanoperedens nitroreducens* and is affiliated to the ANME-2d clade (Raghoebarsing et al., 2006; Haroon et al., 2013). In this study, we report on the environmental genome and transcriptome of a *Methanoperedens*-like archaeon that was found in an enrichment culture performing nitrate-dependent AOM. This draft genome was used to establish a putative model of how nitrate-dependent methanotrophy could work. We discuss how the cytoplasmic process of methane oxidation via reverse methanogenesis may be coupled to the pseudoperiplasmically located reduction of nitrate to nitrite and ammonium by Nar- and Nrf-type nitrogen cycle enzymes. Several cytoplasmic and membrane-bound enzyme complexes homologous to enzymes in methanogens were found and are apparently combined with several metabolic traits not previously found in methanogenic or methanotrophic archaea.

## Materials and methods

### Biological source material

An initial enrichment culture that contained *M. oxyfera* and **A**naerobic oxidation of methane **A**ssociated **A**rchaea (AAA) (Raghoebarsing et al., 2006) was further enriched with the effluent of another reactor that was dominated by *M. oxyfera*. It contained mineral medium saturated with CH_4_, low nitrite (50 μM) and high nitrate (2–3 mM). After about 1 year of enrichment, the reactor was uncoupled from the *M. oxyfera* reactor, and kept flushed with CH_4_-CO_2_ (v:v; 95:5). Nitrate was added daily as sole electron acceptor (1–3 mM final concentration) for over 2 years. The reactor was operated in batch mode. Every 2 weeks, approximately 30% of the supernatant was removed and the reactor replenished with fresh anoxic mineral medium (as previously described by Ettwig et al., 2009, omitting nitrate and nitrite). The AAA microbe in the reactor was closely related to *M. nitroreducens* identified by Haroon et al. (2013) and will subsequently be referred to as *M. nitroreducens* MPEBLZ whereas the strain identified by Haroon et al. will be referred to as *M. nitroreducens* ANME2D.

### Metagenome and -transcriptome sequencing

DNA of the *M. nitroreducens* MPEBLZ enrichment culture was isolated with a method based on bead beating and SDS lysis as described previously (Ettwig et al., 2009). Total RNA was isolated with the Ambion RiboPureTM Bacteria Kit (MO BIO Laboratories, Uden, The Netherlands) according to the manufacturer's manual. DNA and RNA quality was checked by agarose gel electrophoresis, and concentrations were measured in triplicate with the NanoDrop (ND-1000; Isogen Life Science, The Netherlands). All kits used in library preparation and sequencing were obtained from Life technologies (Life Technologies, Carlsbad, CA, USA). Genomic DNA was sheared for 5 min using the Ion Xpress™ Plus Fragment Library Kit. Further library preparation was performed using the Ion Plus Fragment Library Kit following manufacturer's instructions. Size selection of the library was performed using an E-gel 2% agarose gel. The library was used for two sequencing runs. For both runs, emulsion PCR was performed using the OneTouch 200 bp kit and sequencing was performed on an IonTorrent PGM with the Ion PGM 200 bp sequencing kit and an Ion 318 chip, resulting in a total of 10 million reads with an average length of 170 bp. RNA was sequenced after removal of ribosomal RNA using the MicrobExpress kit (Thermo Scientific, Amsterdam, The Netherlands). The library for RNA-seq was prepared using the RNA-seq kit v2 (Life Technologies, Carlsbad, CA, USA) and two sequencing runs were performed as described above for the metagenomic library.

### Assembly, binning, and annotation of the *Methanoperedens nitroreducens* MPEBLZ draft genome

For the construction of the environmental genome, reads were trimmed on quality and length (>100 bp). The remaining 8.1 million reads, average length 196 bp, were assembled *de novo* using the CLC genomics workbench (v6.5.1, CLCbio, Aarhus, Denmark) with word size 31 and bubble size 5000. Contigs were assigned to *M. nitroreducens* MPEBLZ based on coverage and GC content. The obtained 514 contigs were annotated using Prokka (version 1.10, Seemann, 2014) using an additional custom database containing the genomes of methanogens *Methanosarcina barkeri* str. Fusaro (NC_007355), *Methanosarcina mazei* Gö1 (NC_003901) and *Methanosarcina acetivorans* C2A (NC_003552). After annotation, a round of manual curation was performed to correct detected frameshifts and the contigs were re-annotated. Of the total 4528 ORF's identified 2004 were marked as hypothetical proteins after manual curation. CLC genomics workbench and the sequence visualization and annotation tool Artemis was used to analyse the features of annotated contigs (Rutherford et al., 2000). Initially, reference protein sequences belonging to several methanogenic archaea were retrieved in CLC genomics workbench and homologous protein sequences from *M. nitroreducens* MPEBLZ were identified through local BLASTp. Next, BLASTp was used to identify homologs of *M. nitroreducens* target proteins in strain ANME2D. Results were analyzed based on % sequence identity and expectation value (*e*-value). Homologs to strain ANME2D were defined as exhibiting an *e*-value < 10^−10^ and a sequence identity higher than 40%. Signal peptides were predicted with the PRED-SIGNAL tool (Bagos et al., 2009). This Whole Genome Shotgun project has been deposited at DDBJ/EMBL/GenBank under the accession LKCM00000000. The version described in this paper is version LKCM01000000.

### Transcriptome analysis

The draft genome sequence of the *M. nitroreducens* MPEBLZ was used as the template for the transcriptome analysis. Expression analysis was performed with the RNA-Seq Analysis tool from the CLC Genomic Workbench software (version 8.0, CLC-Bio, Aarhus, Denmark) and values are expressed as RPKM [Reads Per Kilobase of exon model per Million mapped reads (Mortazavi et al., 2008)].

### Cofactor analysis of *Methanoperedens nitroreducens* MPEBLZ

For the analysis of lipid soluble electron carriers, reactor biomass was first investigated with fluorescence *in situ* hybridization (FISH) at the time of sampling to quantify the relative amount of *M. nitroreducens*. FISH was performed as described in Daims et al. (2005) with the probes Arch915 and Eubmix (Stahl and Amann, 1991; Daims et al., 1999). About 50% archaea were found with *M. nitroreducens* as the only archaeon present as found by metagenome sequencing. Subsequently, 50 mg freeze dried cells were grinded with a 5/32″ steel ball with a Retsch MM 300 mixer mill at 60 Hz for 2 min. To each of the grinded samples 1 mL water and 500 μL pentane (GC grade) was added. The samples were vortexed for 2 min at maximum speed, placed in an ultrasonic bath for 2 min and centrifuged for 5 min at 12,000 × g. The upper pentane phase was transferred to a new tube. Another 500 μL pentane was added to the lower (water) phase and the extraction procedure was repeated as described above. Both pentane phases were combined and evaporated to dryness under a nitrogen flow. The dried extracts were dissolved in 200 μL methanol/ethanol (80:20) and aliquots of 90 μL were injected on an Agilent 1100 HPLC containing a Merck LiChrospher 100 RP-18 (5 μm) column (250 × 4.6 mm; flow 0.750 mL/min, peak detection by diode array detector [DAD], integration wavelength 248 nm). The DAD was also used to obtain UV/Vis spectra from 200 to 600 nm. After 3 min isocratic elution with 20% ethanol in methanol a linear gradient to 100% ethanol in 12 min followed by 10 min isocratic elution with 100% ethanol was used for separation. Methanol and ethanol were of HPLC grade. Ubiquinone 10 (UQ10, Sigma-Aldrich), menaquinone 4 (MK4, Supelco) and a methanophenazine standard provided by Uwe Deppenmeier (University of Bonn, Germany) were used as reference compounds. Phase contrast and fluorescent micrographs were taken with a Leitz Dialux microscope according to the method by Doddema and Vogels (1978).

## Results and discussion

### Re-construction of the *Methanoperedens nitroreducens* MPEBLZ genome

The here presented genome was reconstructed from a metagenome dataset of a bioreactor enrichment culture that coupled the anaerobic oxidation of methane to nitrate reduction to nitrite and ammonium (Zhu, 2014). Contigs from a *de novo* metagenome assembly were binned based on coverage and GC-content (Supplementary Figure S1). The community in the reactor was dominated by two organisms, *M. nitroreducens* MPEBLZ and an organism closely related to *M. oxyfera* that will not be discussed here (the analysis of all 16S rRNA gene reads of the metagenome is displayed in Supplementary Table 1). The 16S rRNA gene of *M. nitroreducens* MPEBLZ and *M. nitroreducens* ANME2D were 95% identical. Manual curation of the binned contigs resulted in a 3.74 Mb draft genome of *M. nitroreducens* MPEBLZ, on 514 contigs longer than 500 bp. Based on variation present in the reads supporting the draft genome, it is a consensus genome composed of (at least) two very closely related strains. The draft genome contains homologs of all 103 proteins used by Haroon et al. to assess the completeness of the *M. nitroreducens* ANME2D draft genome (Haroon et al., 2013). Additionally, we have assessed completeness of the draft genome using the lineage specific workflow of checkM, resulting in an estimated completeness higher than 96%, based on 228 markers (Parks et al., 2015). Although the coverage of two contigs that encoded the nitrate reductases is higher than that of the contigs containing the core proteins, the sequence composition and gene content of these contigs support their inclusion in the *M. nitroreducens* MPEBLZ draft genome sequence.

### The core pathway of methanotrophy is well conserved and located in the cytoplasm

We found a full reverse methanogenesis pathway in the *M. nitroreducens* MPEBLZ genome which is in accordance with the study of Haroon et al. (2013) and Wang et al. for ANME-2a (Wang et al., 2014). Methane is probably activated by methyl-coenzyme M reductase (Krüger et al., 2003; Shima and Thauer, 2005; Scheller et al., 2010) which was also the most highly transcribed gene cluster detected in the transcriptome. The methyl group is then transferred to the cofactor methanopterin by the action of a Na^+^ translocationg methyl transferase (Hallam et al., 2004). In methanotrophy, this reaction may dissipate part of the membrane potential (Becher et al., 1992); the sodium/proton gradient to drive this reaction has to be built up in the subsequent steps of methanotrophy. After the transfer to methanopterin, the methyl group is oxidized to CO_2_ by the reverse reaction of methanogenic enzymes (Hallam et al., 2004; Scheller et al., 2010; Thauer, 2011). The reverse methanogenesis pathway also contained a *mer* gene which encodes the F_420_-dependent 5, 10-methenyltetrahydromethanopterin reductase. Within the ANME archaea, this gene seems to be confined to members of the ANME-2 clade (Haroon et al., 2013; Wang et al., 2014) and is missing in ANME-1 clade archaea.

We investigated whether biosynthesis pathways for the crucial C1 carrier molecules were encoded in the genome. For the first acceptor of the methyl group, coenzyme M (2-mercaptoethanesulfonate), we observed that the canonical pathway employing the enzymes ComABCDEF was not encoded but instead the pathway as described for Methanosarcinales and Methanomicrobiales that consists of ComDEF together with cysteate synthase (Graham et al., 2009). Both the biosynthesis pathways for methanofuran and methanopterin are not fully resolved in methanogenic archaea; we could however assign putative biosynthesis proteins according to the report of Kaster et al. (2011a) (Supplementary Table 2).

**Figure 1:**
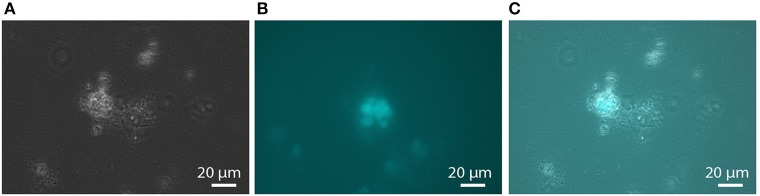
**F_420_ fluorescence of aggregated biomass in the nitrate-AOM enrichment culture**. **(A)** phase contrast micrograph, **(B)** fluorescence micrograph with an excitation wavelength of 390 nm and an emission wavelength of 420 nm, **(C)** overlay of the phase contrast and the fluorescence micrograph showing that not all cells in the aggregates exhibit F_420_ fluorescence.

According to our model, electrons from the core methanotrophic pathway are transferred to the cytoplasmic cofactors F_420_, coenzyme B, and ferredoxin. The biosynthetic pathways for coenzyme B and cofactor F_420_ were, as far as resolved for methanogens (Kaster et al., 2011a), also encoded in the *Methanoperedens* genomes (Supplementary Table 2). Cells sampled from the bioreactor showed typical F_420_ fluorescence as also found in methanogens (Figure 1). The *M. nitroreducens* MPEBLZ genome harbored nine genes encoding soluble [4Fe4S] ferredoxins whereas *Methanosarcina* spp. encode up to 20 (Welte and Deppenmeier, 2011a).

### Nitrate and nitrite reducing enzymes are predicted to be located in the pseudoperiplasm

**Figure 2:**
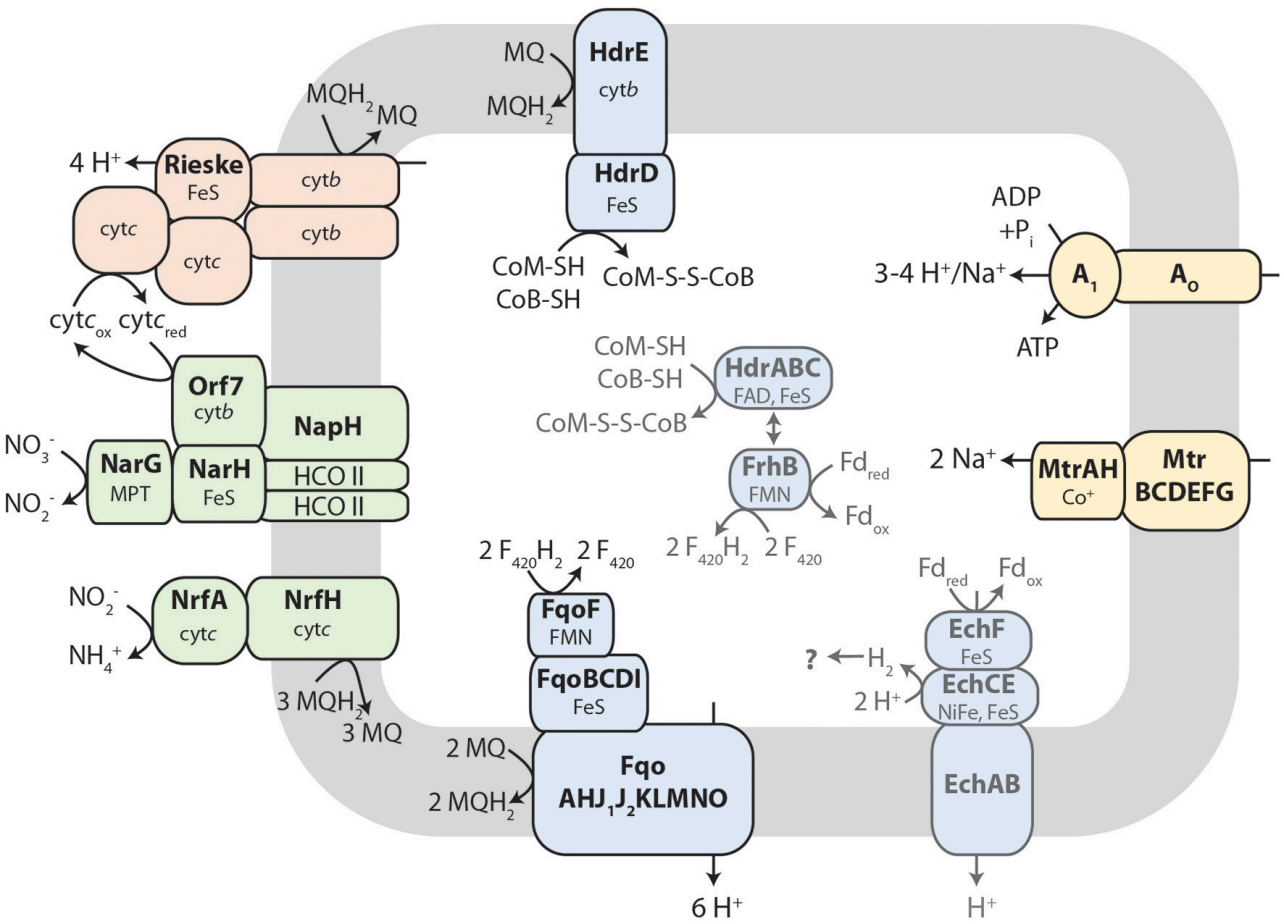
**Tentative metabolic pathway model of membrane-bound electron transport in ***Methanoperedens*****. Reverse methanogenesis produces F_420_H_2_ and the thiol cofactors CoM-SH and CoB-SH as well as reduced ferredoxin (Fd_red_). F_420_H_2_ may be oxidized by the F_420_H_2_ dehydrogenase (Fqo) and electrons transferred to menaquinone (MQ, menaquinone; MQH_2_, menaquinol). The heterodisulfide reductase (Hdr) reaction is reversed resulting in quinone reduction upon CoM-S-S-CoB (heterodisulfide) production. Menaquinol can be oxidized by a Rieske-cytochrome *b* complex comprising two additional cytochrome *c* subunits. Electrons are transferred to an unusual nitrate reductase (Nar) complex, presumably via soluble cytochrome *c* (cyt*c*_ox_/_red_, oxidized/reduced cytochrome *c*), to reduce nitrate to nitrite. A small part of the nitrite can further be reduced to ammonium by nitrite reductase (Nrf) with menaquinol as electron donor. The fate of reduced ferredoxin is unclear. It could either be oxidized by Ech hydrogenase, by FrhB or FqoF (homologous to each other) alone or by the hypothesized confurcating HdrABC-FrhB enzyme complex. For more details, see text. Methyltransferase (Mtr) and A_1_A_O_ ATP synthase make use of the proton motif force built up by the respiratory chain. This metabolic construction is solely based on genome analysis. HCO II, heme copper oxidase subunit II like proteins; cyt*b*, cytochrome *b*; cyt*c*, cytochrome *c*; FeS, iron-sulfur cluster; FMN, flavin mononucleotide; FAD, flavin adenine dinucleotide; MPT, molybdopterin; NiFe, nickel-iron center.

Nitrate reduction in archaea is a process that is not yet well characterized (Martinez-Espinosa et al., 2007). Bacterial Nar-type nitrate reductase has its active site directed toward the cytoplasm, whereas archaeal nitrate reduction by the Nar enzyme seems to take place at the extracellular side of the cytoplasmic membrane (Yoshimatsu et al., 2000; Martinez-Espinosa et al., 2007; de Vries et al., 2010). If, or how, this process is coupled to the build-up of a proton motive force is not yet known. Different protein interaction partners were suggested to anchor the soluble subunits NarGH to the cytoplasmic membrane (Martinez-Espinosa et al., 2007; Yoshimatsu et al., 2007). de Vries et al. (2010) co-purified a protein designated NarM together with NarGH from the *Pyrobaculum aerophilum* membrane fraction and consequently suggested that it forms the membrane anchor spanning the cytoplasmic membrane with one transmembrane helix. It is encoded in an operon with *narGH* and is conserved in most archaeal *nar* operons (de Vries et al., 2010) but not in *M. nitroreducens* MPEBLZ and ANME2D. The *M. nitroreducens* MPEBLZ genome contains two nitrate reductase operons, whereas in the genome of the culture investigated by Haroon et al. (2013) only one was found. One *narG* copy appeared to be part of a conserved gene cluster between the two methanotrophs (MPEBLZ_02035-02041, Table 1) and the respective protein was 24 % identical on amino acid level to the second copy in the *M. nitroreducens* MPEBLZ genome (RPKM value 342). The *narG* operon conserved in the two methanotrophs was further investigated. The gene cluster comprises seven genes with the alpha and beta subunits of the nitrate reductase (NarG and NarH, respectively) encoded in the beginning of the cluster. The NarG protein contains an N-terminal TAT signal peptide for translocation across the cytoplasmic membrane (Table 1); the *M. nitroreducens* genomes also encoded the biosynthetic proteins needed for production and insertion of the molybdopterin cofactor (Supplementary Table 2, Vergnes et al., 2004). In *P. aerophilum* and other archaea, *narGH* are followed by the *narM* gene encoding the putative membrane anchor (de Vries et al., 2010). In *Methanoperedens*, we could not find a homolog to *narM* in the respective *nar* gene cluster or elsewhere in the genome indicating that this organism contains a nitrate reductase with an unusual subunit composition. Other proteins encoded in the gene cluster comprised the chaperone NarJ and a pseudoperiplasmic *b*-type cytochrome homologous to the *Haloferax mediterranei* Orf7 protein which was hypothesized to interact with NarGH in this organism (Martinez-Espinosa et al., 2007). Furthermore, a protein homologous to NapH was encoded in the gene cluster. This protein is a membrane integral subunit with four transmembrane helices of some periplasmic nitrate reductases in bacteria (Brondijk et al., 2002, 2004; Kern and Simon, 2008). It usually co-occurs with NapG that mediates the electron transfer to the catalytic subunits NapAB (Brondijk et al., 2004). In *M. nitroreducens* MPEBLZ and ANME2D, we could not find homologs to NapG or NapAB. The C-terminus of the NapH-like protein is instead extended and contains five additional transmembrane helices. Two other proteins that were encoded in the same gene cluster were homologous to subunit II of heme copper oxidases (Pereira et al., 2001). As all of the genes encoding these proteins were considerably expressed (Table 1) they may form an unusual nitrate reducing (transient) membrane-bound protein complex (Figure 2), a possibility that has to be addressed by further biochemical studies.

**Table 1:** **Description of proteins encoded in the ***nar*** operon of ***Methanoperedens*** species**.

**Locus identifier**	**Homolog to described protein**	**Homolog in ANME2D**	**% identity between ANME2D and MPEBLZ^b^**	**RPKM^c^**	**Transmembrane helices**	**Signal peptide**
MPEBLZ_02035	NarG	ANME2D_03460	81	739	no	1–42
MPEBLZ_02036	NarH	ANME2D_03461	83	685	no	no
MPEBLZ_02037	NarJ	ANME2D_03462	73	792	no	no
MPEBLZ_02038	Orf7	ANME2D_03463	66	1209	no	1–32
MPEBLZ_02039	HCO II^a^	ANME2D_03464	74	630	1	1–23
MPEBLZ_02040	NapH	ANME2D_03465	80	1367	9	no
MPEBLZ_02041	HCO II^a^	ANME2D_03466	65	1396	1	no

a *Heme copper oxidase subunit II*.

b *Alignment covers more than 90% of the query protein sequence, < e^-20^*.

c *Reads per Kilobase per Million mapped reads*.

*All listed proteins have a well conserved homolog in the M. nitroreducens ANME2D genome and the respective genes are highly transcribed in M. nitroreducens MPEBLZ (high RPKM values)*.

In the bioreactor that contained the enriched *M. nitroreducens* MPEBLZ culture, part of the nitrite was further reduced to ammonium (Zhu, 2014). It is evident that not all nitrite was reduced to ammonium as so far *M. nitroreducens* was always co-enriched with dedicated nitrite utilizers like *M. oxyfera* (Haroon et al., 2013; Zhu, 2014) or anaerobic ammonium oxidizers (Haroon et al., 2013) that reduced nitrite to dinitrogen gas. In *Escherichia coli*, the activities of nitrate and nitrite reductases are concerted by a complicated regulatory network (Rabin and Stewart, 1993; Tyson et al., 1993; Chiang et al., 1997; Wang and Gunsalus, 2000; Noriega et al., 2010). Besides the oxygen availability, one of the key factors in *E. coli* for the regulation of transcription of *nar* and *nrf* genes seems to be the concentration of nitrate and nitrite in the culture medium (Wang et al., 1999; Wang and Gunsalus, 2000). In the same studies it was demonstrated that not all nitrite was converted to ammonium under all experimental conditions but is instead excreted into the medium. As a similar finding was observed in the here presented enrichment culture it is probable that also the archaeon *M. nitroreducens* contains regulatory mechanisms for gene expression of nitrogen cycle enzymes.

When we searched the *Methanoperedens* protein complement for enzymes potentially responsible for nitrite-dependent ammonium production, we found proteins that were homologous to the NrfAH type cytochrome *c* nitrite reductase (Figure 2), an enzyme complex that is well characterized in δ- and ε-proteobacteria (Simon et al., 2000; Simon, 2002; Rodrigues et al., 2008). The catalytic subunit NrfA (MPEBLZ_01114, ANME2D_3312) contains—like in bacteria—a signal peptide to be translocated across the cytoplasmic membrane and therefore resides in the archaeal pseudoperiplasm. The amino acid sequence encodes four canonical CxxCH and one CxxCK heme *c* binding motifs for the coordination of five heme *c*. Amino acids required for catalysis and binding of the Ca^2+^ ion were conserved both in MPEBLZ_01114 and the homologous ANME2D_3312 protein [Lys126, Arg106, Tyr216, Gln263, His264, Glu215, Lys 261; *E. coli* NrfA numbering (Bamford et al., 2002)]. The *nrfA* gene is encoded in an operon next to a gene homologous to *nrfH* (MPEBLZ_1115; ANME2D_3311). The corresponding protein NrfH contains the canonical four CxxCH heme *c* binding sites as well as the conserved amino acid residues Lys82 and Asp89. It anchors the NrfAH complex in the cytoplasmic membrane and allows the interaction with the quinone pool. As all amino acids known to be involved in catalysis as well as in cofactor coordination are conserved between the bacterial and the here presented archaeal proteins, and furthermore both proteins were expressed (RPKM value of 102 and 113, respectively), this enzyme complex is the best candidate to catalyze the reduction of nitrite to ammonium also in *Methanoperedens* species.

### Membrane-bound, quinone-dependent electron transport proteins may couple reverse methanogenesis to nitrate reduction

**Figure 3:**
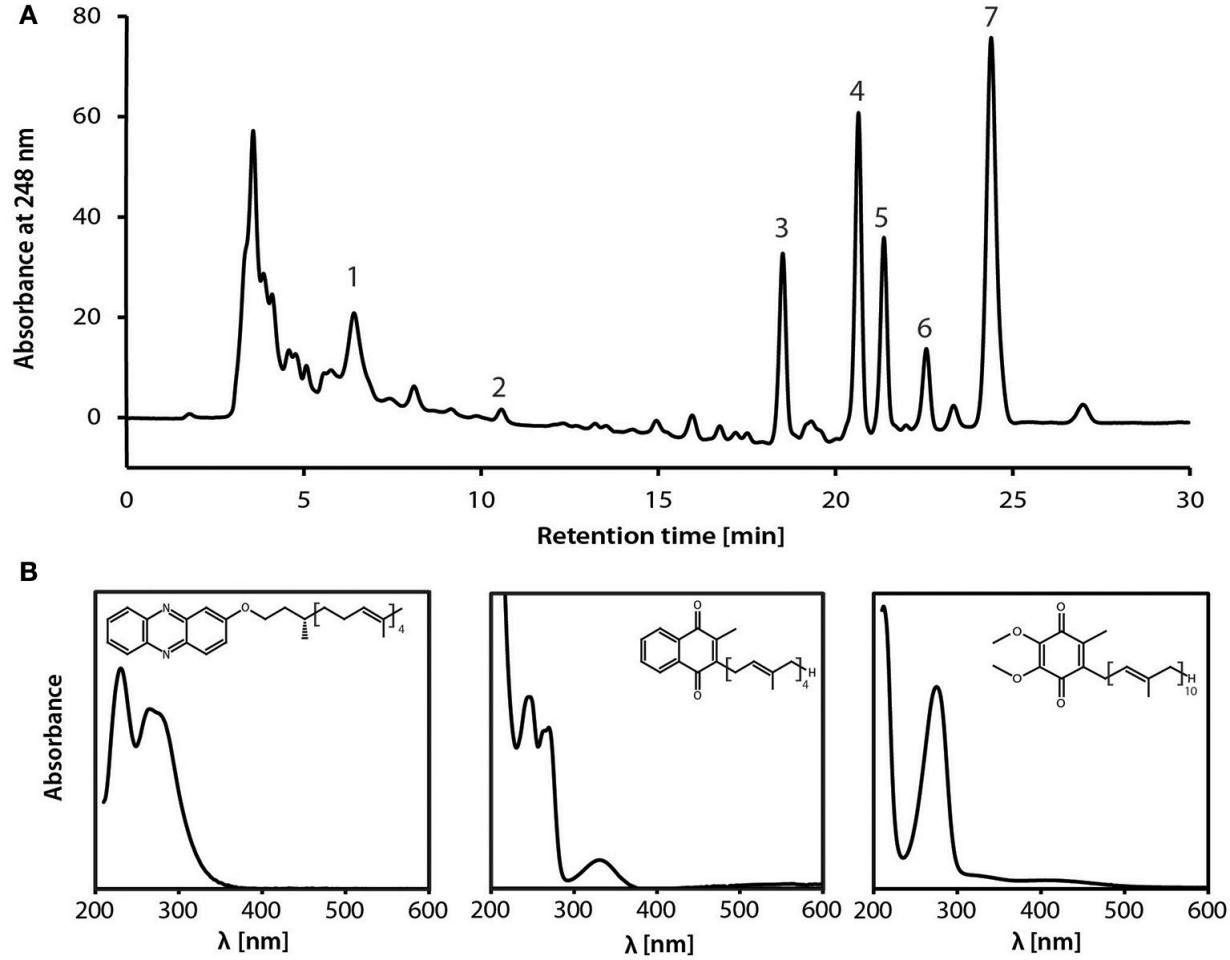
**Analysis of the lipid-soluble electron carriers of the nitrate-AOM enrichment culture**. **(A)** HPLC elution profile as visualized by the absorption at 248 nm. Numbers indicate the peaks that were further characterized by UV/Vis spectroscopy. **(B)** UV-Vis spectra of standard compounds (left, methanophenazine; middle, menaquinone-4; right, ubiquinone-4) for comparison with spectra obtained from fractions separated by HPLC. Based on the comparison of the experimental spectra to the spectra obtained from the HPLC fractions, the different peaks were assigned to contain a representative from the classes of ubiquinones, menaquinones, or methanophenazines. None of the spectra resembled the standard spectrum for methanophenazine (left), seven spectra (obtained from peaks 1, 3, 4, 5, 6, 7,) resembled the standard spectrum of menaquinone-4 (middle) and one spectrum (obtained from peak 2) resembled the standard spectrum of ubiquinone-4. Different retention times within one molecule class indicate a difference in the prenoid chain length. Experimental spectra are displayed in Supplementary Figure S2.

During reverse methanogenesis, electrons are probably transferred to cytoplasmic electron carriers to yield reduced cofactor F_420_ (F_420_H_2_) and reduced ferredoxin. As nitrate reduction seems to take place in the pseudoperiplasm, one of the key questions in generating a metabolic model for nitrate-dependent AOM is how electrons travel across the cytoplasmic membrane and reach the nitrate reductase in the pseudoperiplasm. In the *M. nitroreducens* MPEBLZ genome, we identified several membrane-integral electron transport proteins that may be involved in this process (Figure 2, Supplementary Table 2). We found an F_420_H_2_ dehydrogenase closely related to the F_420_H_2_ dehydrogenase of methanogenic archaea that couples the oxidation of F_420_H_2_ to the build-up of a proton gradient (Welte and Deppenmeier, 2014). All subunits of this complex are well conserved and expressed which strongly indicates that F_420_H_2_ is oxidized by this complex in *Methanoperedens*. The genomic arrangement of the corresponding gene cluster in *Methanoperedens* resembles the one found in *M. mazei*: the F_420_H_2_ interacting subunit FpoF/FqoF is encoded apart from the core F_420_H_2_ dehydrogenase gene cluster at a different location on the chromosome. The F_420_H_2_ dehydrogenase gene cluster also comprises the gene *fpoO* which is only found in Methanosarcinales but for which there is no known function. The F_420_H_2_ dehydrogenase in Methanosarcinales transfers electrons to the membrane integral electron carrier methanophenazine. The enzyme is also found in the related Euryarchaeal lineage Archaeoglobales where a homologous complex mediates electron transport to menaquinone (Kunow et al., 1994; Brüggemann et al., 2000). As methanophenazine (E${}^{0_{{}^{\prime }}}=$ −165 mV, Tietze et al., 2003) and menaquinone (E${}^{0_{{}^{\prime }}}=$ −80 mV, Tran and Unden, 1998) have considerably different redox potentials that have implications for subsequent electron transport pathways in nitrate-dependent AOM, we investigated which of the two lipid-soluble electron acceptors was present in *M. nitroreducens* MPEBLZ. As the biosynthesis pathway for methanophenazine is not known, we could not mine the genome for presence or absence of these genes. In contrast, there is a complete menaquinone biosynthesis pathway known for *Archaeoglobus* (Hemmi et al., 2005; Hiratsuka et al., 2008; Nowicka and Kruk, 2010), which is not present in Methanosarcinales. We found that this pathway was present in both *M. nitroreducens* genomes (Haroon et al., 2013). To obtain further experimental evidence, we extracted quinones and phenazines from the bioreactor biomass that was dominated by cells of strain MPEBLZ and analyzed this fraction with high performance liquid chromatography coupled to UV/Vis spectroscopy (Figure 3). The elution profile of the HPLC chromatogram is shown in Figure 3A. The main peaks in the chromatogram were analyzed with UV/Vis spectroscopy and showed either a ubiquinone-like spectrum (peak 2, comparable to the spectrum in the right panel of Figure 3B) or a menaquinone-like spectrum (peaks 1, 3, 4, 5, 6, 7, comparable to the spectrum in the middle panel of Figure 3B). We could, however, not detect any fraction that showed the characteristic UV/Vis spectrum of methanophenazine (spectrum in the left panel of Figure 3B). As the culture is an enrichment culture it was not possible to assign one of the quinone fractions to *M. nitroreducens* MPEBLZ. From these experiments we conclude that it is highly likely that *M. nitroreducens* MPEBLZ uses menaquinone and not methanophenazine in membrane-bound electron transport (Figure 3).

Besides F_420_H_2_ dehydrogenase, a membrane-bound heterodisulfide reductase (HdrDE, Figure 2) may contribute to the reduction of the quinone pool. In the *M. nitroreducens* MPEBLZ genome we were able to locate the gene for the membrane-integral *b*-type cytochrome subunit HdrE as well as the hydrophilic subunit HdrD. This complex may couple the oxidation of the reduced thiol cofactors CoM-SH and CoB-SH to form the heterodisulfide CoM-S-S-CoB (Figure 2). Electrons may be transferred to the membrane-integral electron carrier menaquinone. As the redox potential E${}^{0_{{}^{\prime }}}$ of the CoM-S-S-CoB/CoM-SH+CoB-SH redox couple is −143 mV (Tietze et al., 2003), electron transfer to menaquinone (E${}^{0_{{}^{\prime }}}=-$80 mV) would be exergonic whereas electron transfer to methanophenazine (E${}^{0_{{}^{\prime }}}=-$165 mV) would be endergonic.

The reduced quinone pool can subsequently be used by membrane-bound oxidoreductases. We could not find membrane-bound quinol oxidases. Instead, we identified an unusual Rieske/cytochrome *b* (Rieske/cyt*b*) complex encoded in the *M. nitroreducens* genomes which is homologous to the cytochrome *bc*_1_ and *b*_6_*f* complexes of chemotrophs and phototrophs, respectively. These complexes couple the oxidation of quinones to the reduction of periplasmic cytochrome *c* and the translocation of protons via the Q-cycle (Berry et al., 2000). The canonical cytochrome *bc*_1_/*b*_6_*f* complex contains a membrane-integral cytochrome *b*, a periplasmic Rieske iron-sulfur protein and another cytochrome (cytochrome *c*_1_ or *f*) at the periplasmic face, all of which are also encoded by the *M. nitroreducens* genome (Supplementary Table 2, MPEBLZ_00818 and MPEBLZ_00820 to 00822). The Rieske/cyt*b* gene cluster also comprises two pentaheme *c*-type cytochromes (MPEBLZ_00816 and 00817) and two hypothetical proteins (MPEBLZ_00819 and 00823), all of which are conserved between the strains MPEBLZ and ANME2D. All eight genes are expressed at similar levels in *M. nitroreducens* MPEBLZ. This transcriptional pattern combined with the gene cluster arrangement suggests that the proteins MPEBLZ_00818 to 00823 may form a non-canonical Rieske/cyt*b* complex which is in line with the finding that those complexes often harbor additional subunits to the canonical ones (Ten Brink et al., 2013). For the related haloarchaea there is strong indication that the Rieske/cyt*b* complex and nitrate reductase are interacting as they are encoded in the same gene cluster (Martinez-Espinosa et al., 2007; Yoshimatsu et al., 2007; Bonete et al., 2008) and the nitrate reductase reaction is inhibited by a the Rieske/cyt*b* complex inhibitor Antimycin A (Martinez-Espinosa et al., 2007). In *M. nitroreducens*, electrons from the reduced quinone pool may travel via the Rieske/cyt*b* complex and the pentaheme *c*-type cytochromes to the pseudoperiplasmic space and at that place they may be used by the nitrate reductase to reduce the external electron acceptor nitrate.

### Ferredoxin and the heterodisulfide may be re-cycled by a novel electron-confurcating enzyme complex

**Figure 4:**
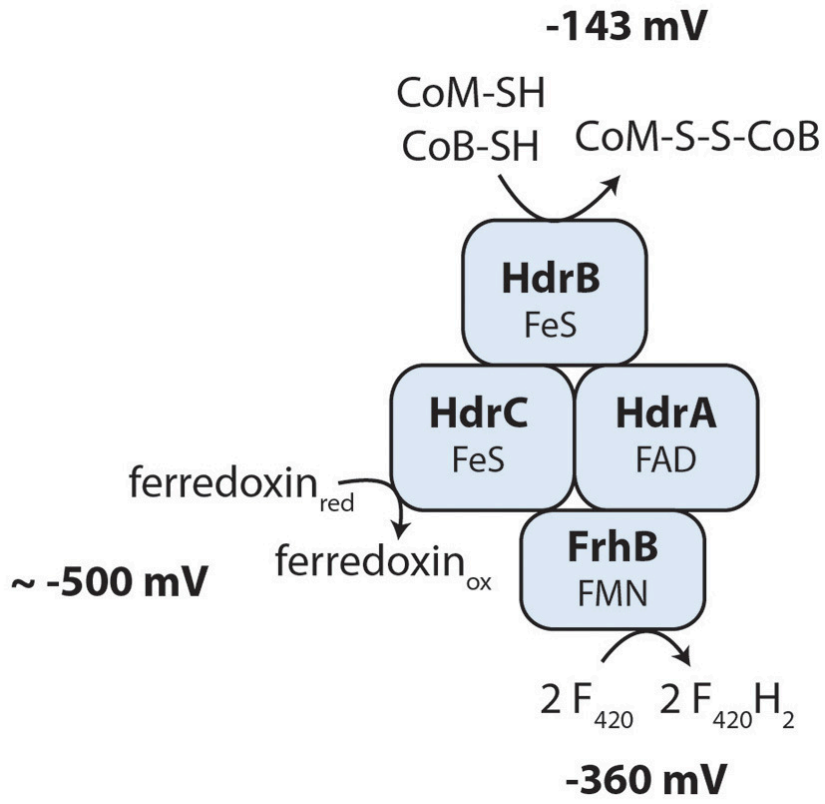
**Hypothesis of a novel cytoplasmic electron confurcating heterodisulfide reductase complex for ferredoxin and CoM-S-S-CoB recycling**. The exergonic reduction of F_420_ (E${}^{0_{{}^{\prime }}}$ = −360 mV) with reduced ferredoxin (E${}^{0_{{}^{\prime }}}$ ≈−500 mV) may be coupled to the endergonic electron transfer from the thiol cofactors CoM-SH and CoB-SH (E${}^{0_{{}^{\prime }}}$ = −143 mV) to F_420_. This hypothesis is based on the metabolic reconstruction using genome and transcriptome sequencing and is not yet supported by biochemical experiments. FAD, flavin adenine dinucleotide; FMN, flavin mononucleotide; FeS, iron-sulfur cluster; FrhB, F_420_-reducing hydrogenase subunit B; HdrABC, heterodisulfide reductase subunits A, B, C.

The oxidized cofactors ferredoxin and heterodisulfide are needed as electron acceptors during reverse methanogenesis and thus have to be re-oxidized by cytoplasmic or membrane-bound electron transport processes to become available for a new round of methane oxidation. Members of the Methanosarcinales use membrane proteins for heterodisulfide reduction and ferredoxin oxidation (Welte and Deppenmeier, 2014). The genome of *Methanoperedens* encodes a membrane-bound heterodisulfide reductase (HdrDE, see above) that is highly expressed (RPKM values 946 and 1396, Supplementary Table 2) and therefore presumably the primary CoM-SH/CoB-SH oxidizing enzyme. For ferredoxin oxidation, we did not find an Rnf complex but identified an Ech hydrogenase lacking the subunit EchD (Supplementary Table 2). In the six-subunit Ech hydrogenase, EchD is the only hydrophilic subunit without prosthetic groups and is missing in Ech hydrogenases of some methanogens (Friedrich and Scheide, 2000). This indicates that the Ech hydrogenase of *Methanoperedens* may be functional. Expression values are, however, low (RPKM 52–80, Supplementary Table 2) so it is unclear whether Ech hydrogenase is used by *M. nitroreducens* under the investigated growth conditions. In hydrogenotrophic methanogens, the cytoplasmic electron bifurcating enzyme complex heterodisulfide reductase (HdrABC) coupled to a hydrogenase (MvhABG) serves as ferredoxin reducing enzyme (Kaster et al., 2011b): electrons from molecular hydrogen are used to reduce the heterodisulfide in an exergonic reaction which in turn drives the endergonic reduction of ferredoxin. When we analyzed the genome for the presence of this enzyme complex, we found that it encodes three copies of *hdrABC* that are significantly expressed (RPKM values 137–482, Supplementary Table 2) but we could not detect the *mvhABG* gene cluster encoding the hydrogenase used in electron bifurcation by methanogenic archaea. In *M. nitroreducens*, the thiols CoM-SH and CoB-SH (E${}^{0_{{}^{\prime }}}=-$143 mV) are produced in reverse methanotrophy and would therefore donate electrons to the HdrABC complex; however, the redox potential is too high to allow for a direct reduction of any of the cytoplasmic electron carriers F_420_, ferredoxin, H_2,_ or NAD(P)^+^. Instead, the oxidation of the CoM-SH and CoB-SH thiols may be coupled to an electron confurcation reaction in which ferredoxin (E${}^{0_{{}^{\prime }}}$ ≈ −500 mV, Thauer et al., 2008) would be oxidized concomitantly and F_420_ (E${}^{0_{{}^{\prime }}}$ = −360 mV, Walsh, 1986) reduced. The overall reaction would turn thermodynamically favorable and allow the backwards electron flow from the CoM-SH and CoB-SH thiols to F_420_. A possible protein mediating the interaction with F_420_ and ferredoxin has been described (FpoF, Welte and Deppenmeier, 2011b) and a gene encoding a homologous protein (FrhB) was found in proximity of one of the *hdrABC* copies, suggesting that an HdrABC-FrhB complex may mediate the above described reaction (Figure 4) in *Methanoperedens* species. FrhB or FpoF alone might also couple the oxidation of ferredoxin to the reduction of F_420_ as observed in the cytoplasm of *M. mazei* (Welte and Deppenmeier, 2011b). In this case, the energy liberated by the redox reaction (ΔE${}^{0_{{}^{\prime }}}$ ≈ 140 mV) would not be harnessed but released as heat.

### Hydrogen does not seem to be an intermediate in the electron transport chain

In many *Methanosarcina* sp., hydrogen is an intermediate in anaerobic respiration. It is produced either by the action of Ech hydrogenase during ferredoxin oxidation or by the action of the cytoplasmic F_420_ hydrogenase (FrhABG) during oxidation of F_420_H_2_ (Meuer et al., 1999; Kulkarni et al., 2009). In both cases, molecular hydrogen is subsequently oxidized by a membrane-bound hydrogenase (VhoACG/VhtACG/VhxACG) to feed electrons into the methanophenazine pool (Welte and Deppenmeier, 2014). In the *M. nitroreducens* MPEBLZ and ANME2D genomes, genes encoding the Vho/Vht/Vhx hydrogenase were not found indicating that electrons from molecular hydrogen cannot enter the membrane-bound anaerobic respiratory chain of *Methanoperedens* species. Furthermore, the *M. nitroreducens* MPEBLZ genome only contained an *frhB* gene but not *frhABG* indicating that the F_420_ hydrogenase is not functional.

### *Methanoperedens nitroreducens* lacks genes required for methanogenesis

As methanotrophic archaea are closely related to methanogens we investigated whether all proteins necessary for one of the methanogenesis pathways were encoded in the genome. For methylotrophic methanogenesis, members of the Methanosarcinales contain substrate-specific methyl transferases (Krzycki, 2004). In the genome of *M. nitroreducens* MPEBLZ we could not find such methyl transferases which makes it unlikely that this species is able to perform methanogenesis from methylated compounds. For hydrogenotrophic methanogenesis, dedicated hydrogenases have to be encoded in the genome (Thauer et al., 2010). For classical hydrogenotrophic methanogenesis, the HdrABC-MvhABG electron bifurcating complex supplies reduced ferredoxin for CO_2_ reduction (Kaster et al., 2011b). As the MvhABG complex was not encoded in the here investigated genome, classical hydrogenotrophic methanogenesis does not seem to be possible. *Methanosarcina* sp. make use of membrane-bound hydrogenases to eventually reduce CO_2_ to CH_4_. The responsible Vho/Vht/Vhx hydrogenase (subunits) could not be found in the *Methanoperedens* genomes which strongly indicates that neither form of hydrogenotrophic methanogenesis is possible.

In case of aceticlastic methanogenesis, acetate first has to be activated to acetyl-CoA. In *Methanosarcina* sp., this happens via acetate kinase and phosphotransacetylase whereas *Methanosaeta* sp. use AMP-dependent acetyl-CoA synthetases (ACS) (Jetten et al., 1992; Welte and Deppenmeier, 2014). In the genome of *M. nitroreducens* MPEBLZ we could not detect genes encoding the first acetate activation system. We found one gene encoding an ADP-dependent ACS. *Methanosaeta* sp. contain several ACS enzymes that are either used in lipid metabolism or aceticlastic methanogenesis; as we only found one ACS enzyme encoded in the *M. nitroreducens* MPEBLZ genome which was more related to those enzymes involved in fatty acid metabolism it is unlikely that *Methanoperedens* archaea can make methane from acetate.

It cannot be excluded that hitherto unknown mechanisms lead to an ability for methanogenesis in *Methanoperedens* species but regarding well investigated metabolic pathways for methane formation this metabolic trait is not likely to be found in these organisms.

### Unusual high number of *c*-Type cytochromes may reflect adaption to the metabolic trait of nitrate reduction

The genomes of *Methanoperedens* species encode an unusually high number of *c*-type cytochromes (Haroon et al., 2013; Kletzin et al., 2015). The occurrence of *c*-type cytochromes in archaea was recently reviewed (Kletzin et al., 2015) and it was highlighted that the ANME-2 clade contains apparently the highest number of *c*-type cytochromes encoded in an archaeal genome. We identified 87 proteins containing at least one CxxCH heme *c* binding motif. Of these 87 proteins, 68 were homologous to proteins found in *M. nitroreducens* ANME2D. Kletzin et al. (2015) analyzed this protein subset and came to the conclusion that only 43 of these 68 were likely to be true *c*-type cytochromes. Nineteen open reading frames in our *M. nitroreducens* MPEBLZ genome assembly encoded CxxCH motif(s) but did not have a homolog in the strain ANME2D. A total of 23 CxxCH motif containing proteins were abundantly expressed (RPKM value >500) and more closely investigated, 18 with homologs in the ANME2D genome and five without (Table 2). The annotation of these proteins gave no indication on their function as they were all annotated either as hypothetical protein or cytochrome *c* protein. Most of the proteins (70%, Table 2) contained three or more CxxCH motifs (even up to 21) and can therefore be regarded as multiheme *c*-type cytochromes. Several of those harbored an additional CxxxCH (MPEBLZ_00816, MPEBLZ_00817, MPEBLZ_03194, MPEBLZ_04300) or CxxxxCH motif (MPEBLZ_01743, MPEBLZ_03195). *c*-type cytochromes generally reside in the periplasm or archaeal pseudoperiplasm (Thöny-Meyer, 1997) and most of the here investigated *c*-type cytochromes contained a putative signal peptide (Table 2) or an N-terminal transmembrane helix (MPEBLZ_01329, MPEBLZ_04299, MPEBLZ_04300). In addition, several of the proteins (e.g., MPEBLZ_00008, MPEBLZ_00016, MPEBLZ_04347) were homologous to each other. Recently, McGlynn and co-workers identified several large multiheme *c*-type cytochromes in ANME archaea that may bridge the S-layer to donate electrons to extracellular partner organisms or metal oxides (Mcglynn et al., 2015). The respective *c*-type cytochromes are (at least in part) conserved in the MPEBLZ genome (MPEBLZ_02503 and 02608) but were hardly expressed under our experimental conditions (RPKM values of 27 and 37, respectively).

**Table 2:** **Analysis of putative ***c***-type cytochromes in the ***M. nitroreducens*** MPEBLZ genome**.

**Locus identifier**	**Number of CxxCH**	**Homolog in ANME2D**	**% identity between ANME2D and MPEBLZ^a^**	**RPKM^c^**	***Ferroglobus placidus* homolog^b^**	**[%] identity over [%] of the query**	***Geoglobus acetivorans* homolog^b^**	**[%] identity over [%] of the query**	**Signal peptide predicted [aa]**
MPEBLZ_04274	4	ANME2D_02830	29	9148	Ferp_0668	38/87	GACE_1846	32/89	1–25
MPEBLZ_04347	1	ANME2D_02824	77	4802	Ferp_0669	56/72	GACE_1846	59/69	1–28
MPEBLZ_00016	4	ANME2D_02827 / _02824	45/43	3683	Ferp_0668	35/44			
MPEBLZ_02042	5	ANME2D_00867	68	1747					1–24
MPEBLZ_01329	5	ANME2D_00431	57	1739	Ferp_1813	54/57	GACE_1361	38/93	
MPEBLZ_01877	2	ANME2D_02837	38 (78%)	1496	Ferp_0670	34/81	GACE_1847	38/91	1–41
MPEBLZ_01878	13	ANME2D_02837	29 (74%)	1472	Ferp_0672	34/87	GACE_1847	47/82	1–21
MPEBLZ_01742	11	ANME2D_00625	32 (54%)	1394	Ferp_0670	32/78	GACE_1847	28/70	
MPEBLZ_00816	5	ANME2D_03235	72	1138					1–28
MPEBLZ_03194	5	ANME2D_00599	71	1073					1–28
MPEBLZ_04299	5	ANME2D_00603	62	993					
MPEBLZ_04340	1	ANME2D_02824	78	932	Ferp_0669	59/71	GACE_1846	55/92	1–28
MPEBLZ_04300	12	ANME2D_00604	73	907	Ferp_1270	34/90	GACE_1341	31/85	
MPEBLZ_01741	21	ANME2D_00625	42	771	Ferp_0670	25/96	GACE_1847	30/97	
MPEBLZ_03195	3	ANME2D_00600	70	767	Ferp_1439	42/94	GACE_0102	39/98	1–24
MPEBLZ_00008	4	ANME2D_02827 / _02824	45/42	610					
MPEBLZ_00817	5	ANME2D_03236	63	578					1–25
MPEBLZ_00818	1	ANME2D_03237	70	560	Ferp_2064	43/66			1–26
MPEBLZ_03918	1	none		1775					1–25
MPEBLZ_01740	21	none		1102					
MPEBLZ_01743	18	none		925	Ferp_0676	30/61	GACE_1847	25/94	1–32
MPEBLZ_01301	1	none		776					
MPEBLZ_01126	2	none		625					1–33

a *Alignment covers more than 90% (otherwise indicated in [%]) of the query protein sequence, < e^-20^*.

b *Closest homolog from respective archaeal proteome with < e^-10^*.

c *Reads per Kilobase per Million mapped reads*.

*Potential cytochrome c binding sites (CxxCH), predicted signal peptides and expression values (RPKM) as well as identity to other archaeal proteins in the NCBI nr database is documented*.

To get insight into the potential function of the 23 abundantly expressed, uncharacterized cytochrome *c* proteins, we looked for homologs in the nr database using BLASTp (Altschul et al., 1990). For the majority of proteins, no close homologs (besides in strain ANME2D) could be found. An exception to this formed MPEBLZ_02042 which had many homologs in the bacterial and archaeal domain albeit with only moderate sequence identity (= 37%). Homologs that were found for many of the other proteins comprised proteins from the Fe(III) reducing Euryarchaeota *Ferroglobus placidus* and *Geoglobus acetivorans* (Mardanov et al., 2015; Smith et al., 2015). The sequence identity was generally low and spanned only part of the protein (Table 2). For both the proteins from *F. placidus* as well as *G. acetivorans*, there are no biochemical data available as to their function. However, a recent genomic survey (Mardanov et al., 2015) proposed a potential involvement of several *c*-type cytochromes in Fe(III) reduction which were encoded by four gene clusters (gace_0099 to 0102 and gace_1341 to _1344, gace_1360 to _1361, gace_1843 to _1847). Many of the *c*-type cytochromes identified in the *Methanoperedens* genomes showed homology to proteins encoded in the first two gene clusters. The identity of the amino acid sequences was too weak to allow for a functional comparison between the proteins but it indicates that these proteins (which are also highly expressed) may have a function in pseudoperiplasmic electron transport, possibly to nitrate reductase that resides in the pseudoperiplasm. The protein GACE_1847 is speculated to be the direct electron transfer protein from the cytoplasmic membrane to extracellular hematite crystals in *G. acetivorans* (Fe_2_O_3_, Mardanov et al., 2015). It seems to be anchored in the cytoplasmic membrane, span the pseudoperiplasm with an array of *c*-type hemes (16 CxxCH binding motifs) and then bridge the S-layer to make contact to hematite crystals (Fe_2_O_3_) making use of hematite binding motifs at the C-terminus of the protein ([ST]-[AVILMFYW]-[ST]-P-[ST], Lower et al., 2008; Mardanov et al., 2015). The *Methanoperedens* homologs, however, lack the C-terminal amino acid sequence, both for crossing of the S-layer as well as the hematite binding motifs. It is thus unlikely that these proteins are involved in binding to Fe(III) minerals.

Three of the *c*-type cytochrome encoding genes (MPEBLZ_00816 to MPEBLZ_00818) were in the same gene cluster as other subunits for a Rieske/cyt*b* complex indicating their involvement in electron transport from the cytoplasm to the pseudoperiplasm where nitrate and nitrite reductases reside.

The metabolically and phylogenetically closely related methanogens of the order Methanosarcinales only contain few *c*-type cytochromes whose functions are largely unknown. The surprisingly large number of *c*-type cytochromes encoded by ANME-2d archaea may thus be connected to electron transfer from reverse methanogenesis to nitrate reductase. This is in accordance with the anticipated redox potentials of part of these cytochromes and the apparent low number of encoded ferredoxins in the genome: whereas bioenergetics in methanogenic archaea spans the redox range from about −500 mV (E${}^{0_{{}^{\prime }}}$ for CO_2_/CO and at the same time midpoint potential of ferredoxins used in this process) to −143 mV (E${}^{0_{{}^{\prime }}}$ (CoM-S-S-CoB/CoM-SH+CoB-SH)), nitrate-dependent methanotrophic archaea operate at a wider redox potential range (E${}^{0_{{}^{\prime }}}$ (NO${}_{3}^{-}$/NO${}_{2}^{-}$) = +433 mV) that requires electron carriers with a redox potential of up to +400 mV. *c*-type cytochromes are ideal candidates to operate in the redox potential range of −143 to +433 mV and therefore act as redox carriers in nitrate-dependent anaerobic methanotrophy.

### Bioenergetics of nitrate-dependent AOM

The free energy change associated to nitrate-dependent AOM is high with a Gibbs free energy of ΔG^0^' = −523 kJ per mol CH_4_ oxidized [calculated with the standard potentials E${}^{0_{{}^{\prime }}}$ (NO${}_{3}^{-}$/NO${}_{2}^{-}$) = +433 mV and E${}^{0_{{}^{\prime }}}$ (CO_2_/CH_4_) = −244 mV (Thauer et al., 1977)]. According to our metabolic reconstruction (Figure 2), the mechanism of energy conservation in *Methanoperedens* is electron transport phosphorylation and not substrate level phosphorylation. During reverse methanogenesis, 2 mol Na^+^ per mol of methane are translocated into the cytoplasm and dissipate the proton/sodium motive force. At the same time, electrons are transferred to the cytoplasmic cofactors F_420_, ferredoxin and CoM-S-S-CoB. F_420_H_2_ is recycled via the membrane-bound F_420_H_2_ dehydrogenase complex and electrons are transferred to a membrane-bound electron carrier, probably menaquinone. In *Ms. mazei*, two protons are translocated across the membrane in the course of this reaction (Bäumer et al., 2000); as *Methanoperedens* probably uses the more electropositive menaquinone instead of methanophenazine this reaction yields a higher ΔE and subsequently may catalyze the translocation of up to 3 H^+^/2 e^−^. Quinols may be oxidized by the Rieske/cyt*b* complex that transfers electrons to pseudoperiplasmic cytochrome *c*; in the course of this reaction, usually 4 H^+^/2 e^−^ are released at the extracellular side of the membrane via a Q-cycle mechanism (Berry et al., 2000). As for CoM-SH+CoB-SH and ferredoxin also cytoplasmic possibilities to be re-oxidized exist (Figure 4) it is unclear whether their re-oxidation is associated to a build-up of proton motive force. For nitrate reduction in the pseudoperiplasm, it is also not known whether this process is associated to the membrane and thus could contribute to the generation of a proton motive force. Taking together the above mentioned observations, it becomes clear that the oxidation of the four electrons of F_420_H_2_ reduced during reverse methanogenesis leads to proton translocation that by far compensates for the 2 Na^+^ per mol methane that are imported during reverse methanogenesis and allows for the build-up of a proton motive force. This can in turn be used by an A_1_A_O_ ATP synthase to produce ATP for cellular metabolism and growth. The c-subunit of the *M. nitroreducens* ATP synthase (MPEBLZ_01699) resembles the *M. acetivorans* c-subunit; all residues required for Na^+^ and H^+^ binding are conserved (except for the replacement of Thr-67 by Ser which is a common feature of Na^+^ translocating ATP synthases) and may therefore drive ATP synthesis by both a proton and a sodium ion gradient (Schlegel et al., 2012).

## Author contributions

CW, HO, and MJ designed research. AA, DS, and RD collected data and all authors performed parts of the data analysis. CW wrote the paper with contributions from all authors.

### Conflict of interest statement

The authors declare that the research was conducted in the absence of any commercial or financial relationships that could be construed as a potential conflict of interest.

## References

[B1] Altschul S. F. Gish W. Miller W. Myers E. W. Lipman D. J. (1990). Basic local alignment search tool. J. Mol. Biol. 215, 403–410. 10.1016/S0022-2836(05)80360-2 2231712

[B2] Bagos P. G. Tsirigos K. D. Plessas S. K. Liakopoulos T. D. Hamodrakas S. J. (2009). Prediction of signal peptides in archaea. Protein Eng. Des. Sel. 22, 27–35. 10.1093/protein/gzn064 18988691

[B3] Bamford V. A. Angove H. C. Seward H. E. Thomson A. J. Cole J. A. Butt J. N.. (2002). Structure and spectroscopy of the periplasmic cytochrome *c* nitrite reductase from *Escherichia coli*. Biochemistry 41, 2921–2931. 10.1021/bi015765d 11863430

[B4] Bäumer S. Ide T. Jacobi C. Johann A. Gottschalk G. Deppenmeier U. (2000). The F_420_H_2_ dehydrogenase from *Methanosarcina mazei* is a redox-driven proton pump closely related to NADH dehydrogenases. J. Biol. Chem. 275, 17968–17973. 10.1074/jbc.M000650200 10751389

[B5] Becher B. Müller V. Gottschalk G. (1992). N^5^-methyl-tetrahydromethanopterin:coenzyme M methyltransferase of *Methanosarcina strain* Gö1 is an Na^+^-translocating membrane protein. J. Bacteriol. 174, 7656–7660. 1447136 10.1128/jb.174.23.7656-7660.1992 PMC207478

[B6] Berry E. A. Guergova-Kuras M. Huang L. S. Crofts A. R. (2000). Structure and function of cytochrome *bc* complexes. Annu. Rev. Biochem. 69, 1005–1075. 10.1146/annurev.biochem.69.1.1005 10966481

[B7] Boetius A. Ravenschlag K. Schubert C. J. Rickert D. Widdel F. Gieseke A.. (2000). A marine microbial consortium apparently mediating anaerobic oxidation of methane. Nature 407, 623–626. 10.1038/35036572 11034209

[B8] Bonete M. J. Martinez-Espinosa R. M. Pire C. Zafrilla B. Richardson D. J. (2008). Nitrogen metabolism in haloarchaea. Saline Syst. 4:9. 10.1186/1746-1448-4-9 18593475 PMC2483277

[B9] Brondijk T. H. Fiegen D. Richardson D. J. Cole J. A. (2002). Roles of NapF, NapG and NapH, subunits of the *Escherichia coli* periplasmic nitrate reductase, in ubiquinol oxidation. Mol. Microbiol. 44, 245–255. 10.1046/j.1365-2958.2002.02875.x 11967083

[B10] Brondijk T. H. Nilavongse A. Filenko N. Richardson D. J. Cole J. A. (2004). NapGH components of the periplasmic nitrate reductase of *Escherichia coli* K-12: location, topology and physiological roles in quinol oxidation and redox balancing. Biochem. J. 379, 47–55. 10.1042/bj20031115 14674886 PMC1224043

[B11] Brüggemann H. Falinski F. Deppenmeier U. (2000). Structure of the F_420_H_2_: quinone oxidoreductase of *Archaeoglobus fulgidus* - Identification and overproduction of the F_420_H_2_-oxidizing subunit. Eur. J. Biochem. 267, 5810–5814. 10.1046/j.1432-1327.2000.01657.x 10971593

[B12] Chiang R. C. Cavicchioli R. Gunsalus R. P. (1997). ‘Locked-on’ and ‘locked-off’ signal transduction mutations in the periplasmic domain of the *Escherichia coli* NarQ and NarX sensors affect nitrate- and nitrite-dependent regulation by NarL and NarP. Mol. Microbiol. 24, 1049–1060. 10.1046/j.1365-2958.1997.4131779.x 9220011

[B13] Daims H. Bruhl A. Amann R. Schleifer K. H. Wagner M. (1999). The domain-specific probe EUB338 is insufficient for the detection of all *Bacteria*: development and evaluation of a more comprehensive probe set. Syst. Appl. Microbiol. 22, 434–444. 10.1016/S0723-2020(99)80053-8 10553296

[B14] Daims H. Stoecker K. Wagner M. (2005). Fluorescence *in situ* hybridization for the detection of prokaryotes, in Molecular Microbial Ecology, eds Osborn A. M. Smith C. J. (New York, NY: Taylor & Francis Group), 213–239.

[B15] de Vries S. Momcilovic M. Strampraad M. J. Whitelegge J. P. Baghai A. Schroder I. (2010). Adaptation to a high-tungsten environment: *Pyrobaculum aerophilum* contains an active tungsten nitrate reductase. Biochemistry 49, 9911–9921. 10.1021/bi100974v 20863064

[B16] Doddema H. J. Vogels G. D. (1978). Improved identification of methanogenic bacteria by fluorescence microscopy. Appl. Environ. Microbiol. 36, 752–754. 103504 10.1128/aem.36.5.752-754.1978 PMC243133

[B17] Ettwig K. F. Butler M. K. Le Paslier D. Pelletier E. Mangenot S. Kuypers M. M.. (2010). Nitrite-driven anaerobic methane oxidation by oxygenic bacteria. Nature 464, 543–548. 10.1038/nature08883 20336137

[B18] Ettwig K. F. Speth D. R. Reimann J. Wu M. L. Jetten M. S. Keltjens J. T. (2012). Bacterial oxygen production in the dark. Front. Microbiol. 3:273. 10.3389/fmicb.2012.00273 22891064 PMC3413370

[B19] Ettwig K. F. Van Alen T. Van De Pas-Schoonen K. T. Jetten M. S. Strous M. (2009). Enrichment and molecular detection of denitrifying methanotrophic bacteria of the NC10 phylum. Appl. Environ. Microbiol. 75, 3656–3662. 10.1128/AEM.00067-09 19329658 PMC2687271

[B20] Friedrich T. Scheide D. (2000). The respiratory complex I of bacteria, archaea and eukarya and its module common with membrane-bound multisubunit hydrogenases. FEBS Lett. 479, 1–5. 10.1016/S0014-5793(00)01867-6 10940377

[B21] Graham D. E. Taylor S. M. Wolf R. Z. Namboori S. C. (2009). Convergent evolution of coenzyme M biosynthesis in the *Methanosarcinales*: cysteate synthase evolved from an ancestral threonine synthase. Biochem. J. 424, 467–478. 10.1042/BJ20090999 19761441

[B22] Hallam S. J. Putnam N. Preston C. M. Detter J. C. Rokhsar D. Richardson P. M.. (2004). Reverse methanogenesis: testing the hypothesis with environmental genomics. Science 305, 1457–1462. 10.1126/science.1100025 15353801

[B23] Haroon M. F. Hu S. Shi Y. Imelfort M. Keller J. Hugenholtz P.. (2013). Anaerobic oxidation of methane coupled to nitrate reduction in a novel archaeal lineage. Nature 500, 567–570. 10.1038/nature12375 23892779

[B24] Hemmi H. Takahashi Y. Shibuya K. Nakayama T. Nishino T. (2005). Menaquinone-specific prenyl reductase from the hyperthermophilic archaeon *Archaeoglobus fulgidus*. J. Bacteriol. 187, 1937–1944. 10.1128/JB.187.6.1937-1944.2005 15743940 PMC1064032

[B25] Hiratsuka T. Furihata K. Ishikawa J. Yamashita H. Itoh N. Seto H.. (2008). An alternative menaquinone biosynthetic pathway operating in microorganisms. Science 321, 1670–1673. 10.1126/science.1160446 18801996

[B26] Jetten M. S. M. Stams A. J. M. Zehnder A. J. B. (1992). Methanogenesis from acetate - a comparison of the acetate metabolism in *Methanothrix soehngenii* and *Methanosarcina* spp. FEMS Microbiol. Rev. 88, 181–197. 10.1111/j.1574-6968.1992.tb04987.x

[B27] Kaster A. K. Goenrich M. Seedorf H. Liesegang H. Wollherr A. Gottschalk G.. (2011a). More than 200 genes required for methane formation from H(2) and CO(2) and energy conservation are present in *Methanothermobacter marburgensis* and *Methanothermobacter thermautotrophicus*. Archaea 2011:973848. 10.1155/2011/973848 21559116 PMC3087415

[B28] Kaster A. K. Moll J. Parey K. Thauer R. K. (2011b). Coupling of ferredoxin and heterodisulfide reduction via electron bifurcation in hydrogenotrophic methanogenic archaea. Proc. Natl. Acad. Sci. U.S.A. 108, 2981–2986. 10.1073/pnas.1016761108 21262829 PMC3041090

[B29] Kern M. Simon J. (2008). Characterization of the NapGH quinol dehydrogenase complex involved in *Wolinella succinogenes* nitrate respiration. Mol. Microbiol. 69, 1137–1152. 10.1111/j.1365-2958.2008.06361.x 18631238

[B30] Kletzin A. Heimerl T. Flechsler J. van Niftrik L. Rachel R. Klingl A. (2015). Cytochromes *c* in *Archaea*: distribution, maturation, cell architecture, and the special case of *Ignicoccus hospitalis*. Front. Microbiol. 6:439. 10.3389/fmicb.2015.00439 26029183 PMC4429474

[B31] Knittel K. Boetius A. (2009). Anaerobic oxidation of methane: progress with an unknown process. Annu. Rev. Microbiol. 63, 311–334. 10.1146/annurev.micro.61.080706.093130 19575572

[B32] Krüger M. Meyerdierks A. Glöckner F. O. Amann R. Widdel F. Kube M.. (2003). A conspicuous nickel protein in microbial mats that oxidize methane anaerobically. Nature 426, 878–881. 10.1038/nature02207 14685246

[B33] Krzycki J. A. (2004). Function of genetically encoded pyrrolysine in corrinoid-dependent methylamine methyltransferases. Curr. Opin. Chem. Biol. 8, 484–491. 10.1016/j.cbpa.2004.08.012 15450490

[B34] Kulkarni G. Kridelbaugh D. M. Guss A. M. Metcalf W. W. (2009). Hydrogen is a preferred intermediate in the energy-conserving electron transport chain of *Methanosarcina barkeri*. Proc. Natl. Acad. Sci. U.S.A. 106, 15915–15920. 10.1073/pnas.0905914106 19805232 PMC2747218

[B35] Kunow J. Linder D. Stetter K. O. Thauer R. K. (1994). F_420_H_2_:quinone oxidoreductase from *Archaeoglobus fulgidus* - characterization of a membrane-bound multisubunit complex containing FAD and iron-sulfur clusters. Eur. J. Biochem. 223, 503–511. 10.1111/j.1432-1033.1994.tb19019.x 8055920

[B36] Lower B. H. Lins R. D. Oestreicher Z. Straatsma T. P. Hochella M. F. Jr. Shi L.. (2008). *In vitro* evolution of a peptide with a hematite binding motif that may constitute a natural metal-oxide binding archetype. Environ. Sci. Technol. 42, 3821–3827. 10.1021/es702688c 18546729

[B37] Mardanov A. V. Slododkina G. B. Slobodkin A. I. Beletsky A. V. Gavrilov S. N. Kublanov I. V.. (2015). The *Geoglobus acetivorans* genome: Fe(III) reduction, acetate utilization, autotrophic growth, and degradation of aromatic compounds in a hyperthermophilic archaeon. Appl. Environ. Microbiol. 81, 1003–1012. 10.1128/AEM.02705-14 25416759 PMC4292469

[B38] Martinez-Espinosa R. M. Dridge E. J. Bonete M. J. Butt J. N. Butler C. S. Sargent F.. (2007). Look on the positive side! The orientation, identification and bioenergetics of ‘Archaeal’ membrane-bound nitrate reductases. FEMS Microbiol. Lett. 276, 129–139. 10.1111/j.1574-6968.2007.00887.x 17888006

[B39] Mcglynn S. E. Chadwick G. L. Kempes C. P. Orphan V. J. (2015). Single cell activity reveals direct electron transfer in methanotrophic consortia. Nature 526, 531–535. 10.1038/nature15512 26375009

[B40] Metcalf W. W. Griffin B. M. Cicchillo R. M. Gao J. Janga S. C. Cooke H. A.. (2012). Synthesis of methylphosphonic acid by marine microbes: a source for methane in the aerobic ocean. Science 337, 1104–1107. 10.1126/science.1219875 22936780 PMC3466329

[B41] Meuer J. Bartoschek S. Koch J. Künkel A. Hedderich R. (1999). Purification and catalytic properties of Ech hydrogenase from *Methanosarcina barkeri*. Eur. J. Biochem. 265, 325–335. 10.1046/j.1432-1327.1999.00738.x 10491189

[B42] Mortazavi A. Williams B. A. Mccue K. Schaeffer L. Wold B. (2008). Mapping and quantifying mammalian transcriptomes by RNA-Seq. Nat. Methods 5, 621–628. 10.1038/nmeth.1226 18516045 PMC13303166

[B43] Noriega C. E. Lin H. Y. Chen L. L. Williams S. B. Stewart V. (2010). Asymmetric cross-regulation between the nitrate-responsive NarX-NarL and NarQ-NarP two-component regulatory systems from *Escherichia coli* K-12. Mol. Microbiol. 75, 394–412. 10.1111/j.1365-2958.2009.06987.x 19968795 PMC3034140

[B44] Nowicka B. Kruk J. (2010). Occurrence, biosynthesis and function of isoprenoid quinones. Biochim. Biophys. Acta 1797, 1587–1605. 10.1016/j.bbabio.2010.06.007 20599680

[B45] Parks D. H. Imelfort M. Skennerton C. T. Hugenholtz P. Tyson G. W. (2015). CheckM: assessing the quality of microbial genomes recovered from isolates, single cells, and metagenomes. Genome Res. 25, 1043–1055. 10.1101/gr.186072.114 25977477 PMC4484387

[B46] Pereira M. M. Santana M. Teixeira M. (2001). A novel scenario for the evolution of haem-copper oxygen reductases. Biochim. Biophys. Acta 1505, 185–208. 10.1016/S0005-2728(01)00169-4 11334784

[B47] Rabin R. S. Stewart V. (1993). Dual response regulators (NarL and NarP) interact with dual sensors (NarX and NarQ) to control nitrate- and nitrite-regulated gene expression in *Escherichia coli* K-12. J. Bacteriol. 175, 3259–3268. 8501030 10.1128/jb.175.11.3259-3268.1993 PMC204722

[B48] Raghoebarsing A. A. Pol A. van de Pas-Schoonen K. T. Smolders A. J. Ettwig K. F. Rijpstra W. I.. (2006). A microbial consortium couples anaerobic methane oxidation to denitrification. Nature 440, 918–921. 10.1038/nature04617 16612380

[B49] Rodrigues M. L. Scott K. A. Sansom M. S. Pereira I. A. Archer M. (2008). Quinol oxidation by *c*-type cytochromes: structural characterization of the menaquinol binding site of NrfHA. J. Mol. Biol. 381, 341–350. 10.1016/j.jmb.2008.05.066 18597779

[B50] Ruff S. E. Biddle J. F. Teske A. P. Knittel K. Boetius A. Ramette A. (2015). Global dispersion and local diversification of the methane seep microbiome. Proc. Natl. Acad. Sci. U.S.A. 112, 4015–4020. 10.1073/pnas.1421865112 25775520 PMC4386351

[B51] Rutherford K. Parkhill J. Crook J. Horsnell T. Rice P. Rajandream M. A.. (2000). Artemis: sequence visualization and annotation. Bioinformatics 16, 944–945. 10.1093/bioinformatics/16.10.944 11120685

[B52] Scheller S. Goenrich M. Boecher R. Thauer R. K. Jaun B. (2010). The key nickel enzyme of methanogenesis catalyses the anaerobic oxidation of methane. Nature 465, 606–608. 10.1038/nature09015 20520712

[B53] Schlegel K. Leone V. Faraldo-Gómez J. D. Müller V. (2012). Promiscuous archaeal ATP synthase concurrently coupled to Na^+^ and H^+^ translocation. Proc. Natl. Acad. Sci. U.S.A. 109, 947–952. 10.1073/pnas.1115796109 22219361 PMC3271924

[B54] Seemann T. (2014). Prokka: rapid prokaryotic genome annotation. Bioinformatics 30, 2068–2069. 10.1093/bioinformatics/btu153 24642063

[B55] Shima S. Thauer R. K. (2005). Methyl-coenzyme M reductase and the anaerobic oxidation of methane in methanotrophic Archaea. Curr. Opin. Microbiol. 8, 643–648. 10.1016/j.mib.2005.10.002 16242993

[B56] Simon J. (2002). Enzymology and bioenergetics of respiratory nitrite ammonification. FEMS Microbiol. Rev. 26, 285–309. 10.1111/j.1574-6976.2002.tb00616.x 12165429

[B57] Simon J. Gross R. Einsle O. Kroneck P. M. Kroger A. Klimmek O. (2000). A NapC/NirT-type cytochrome *c* (NrfH) is the mediator between the quinone pool and the cytochrome *c* nitrite reductase of *Wolinella succinogenes*. Mol. Microbiol. 35, 686–696. 10.1046/j.1365-2958.2000.01742.x 10672190

[B58] Sirajuddin S. Rosenzweig A. C. (2015). Enzymatic oxidation of methane. Biochemistry 54, 2283–2294. 10.1021/acs.biochem.5b00198 25806595 PMC5257249

[B59] Smith J. A. Aklujkar M. Risso C. Leang C. Giloteaux L. Holmes D. E. (2015). Mechanisms involved in Fe(III) respiration by the hyperthermophilic archaeon *Ferroglobus placidus*. Appl. Environ. Microbiol. 81, 2735–2744. 10.1128/AEM.04038-14 25662973 PMC4375341

[B60] Stahl D. A. Amann R. (1991). Development and application of nucleic acid probes in bacterial systematics, in Sequencing and Hybridization Techniques in Bacterial Systematics, eds Stackebrandt E. Goodfellow M. (Chichester: John Wiley & Sons), 205–248.

[B61] Ten Brink F. Schoepp-Cothenet B. van Lis R. Nitschke W. Baymann F. (2013). Multiple Rieske/cyt*b* complexes in a single organism. Biochim. Biophys. Acta 1827, 1392–1406. 10.1016/j.bbabio.2013.03.003 23507620

[B62] Thauer R. K. (2011). Anaerobic oxidation of methane with sulfate: on the reversibility of the reactions that are catalyzed by enzymes also involved in methanogenesis from CO_2_. Curr. Opin. Microbiol. 14, 292–299. 10.1016/j.mib.2011.03.003 21489863

[B63] Thauer R. K. Jungermann K. Decker K. (1977). Energy conservation in chemotrophic anaerobic bacteria. Bacteriol. Rev. 41, 100–180. 860983 10.1128/br.41.1.100-180.1977 PMC413997

[B64] Thauer R. K. Kaster A. K. Goenrich M. Schick M. Hiromoto T. Shima S. (2010). Hydrogenases from methanogenic archaea, nickel, a novel cofactor, and H_2_ storage. Annu. Rev. Biochem. 79, 507–536. 10.1146/annurev.biochem.030508.152103 20235826

[B65] Thauer R. K. Kaster A. K. Seedorf H. Buckel W. Hedderich R. (2008). Methanogenic archaea: ecologically relevant differences in energy conservation. Nat. Rev. Microbiol. 6, 579–591. 10.1038/nrmicro1931 18587410

[B66] Thöny-Meyer L. (1997). Biogenesis of respiratory cytochromes in bacteria. Microbiol. Mol. Biol. Rev. 61, 337–376. 9293186 10.1128/mmbr.61.3.337-376.1997 PMC232615

[B67] Tietze M. Beuchle A. Lamla I. Orth N. Dehler M. Greiner G.. (2003). Redox potentials of methanophenazine and CoB-S-S-CoM, factors involved in electron transport in methanogenic archaea. Chembiochem 4, 333–335. 10.1002/cbic.200390053 12672112

[B68] Tran Q. H. Unden G. (1998). Changes in the proton potential and the cellular energetics of *Escherichia coli* during growth by aerobic and anaerobic respiration or by fermentation. Eur. J. Biochem. 251, 538–543. 10.1046/j.1432-1327.1998.2510538.x 9492330

[B69] Tyson K. L. Bell A. I. Cole J. A. Busby S. J. (1993). Definition of nitrite and nitrate response elements at the anaerobically inducible *Escherichia coli nirB* promoter: interactions between FNR and NarL. Mol. Microbiol. 7, 151–157. 10.1111/j.1365-2958.1993.tb01106.x 8437517

[B70] Vergnes A. Gouffi-Belhabich K. Blasco F. Giordano G. Magalon A. (2004). Involvement of the molybdenum cofactor biosynthetic machinery in the maturation of the *Escherichia coli* nitrate reductase A. J. Biol. Chem. 279, 41398–41403. 10.1074/jbc.M407087200 15247236

[B71] Walsh C. (1986). Naturally occurring 5-deazaflavin coenzymes - biological redox roles. Acc. Chem. Res. 19, 216–221. 10.1021/ar00127a004

[B72] Wang F. P. Zhang Y. Chen Y. He Y. Qi J. Hinrichs K. U.. (2014). Methanotrophic archaea possessing diverging methane-oxidizing and electron-transporting pathways. ISME J. 8, 1069–1078. 10.1038/ismej.2013.212 24335827 PMC3996691

[B73] Wang H. Gunsalus R. P. (2000). The *nrfA* and *nirB* nitrite reductase operons in *Escherichia coli* are expressed differently in response to nitrate than to nitrite. J. Bacteriol. 182, 5813–5822. 10.1128/JB.182.20.5813-5822.2000 11004182 PMC94705

[B74] Wang H. Tseng C. P. Gunsalus R. P. (1999). The *napF* and *narG* nitrate reductase operons in *Escherichia coli* are differentially expressed in response to submicromolar concentrations of nitrate but not nitrite. J. Bacteriol. 181, 5303–5308. 10464201 10.1128/jb.181.17.5303-5308.1999 PMC94036

[B75] Welte C. Deppenmeier U. (2011a). Membrane-bound electron transport in *Methanosaeta thermophila*. J. Bacteriol. 193, 2868–2870. 10.1128/JB.00162-11 21478356 PMC3133127

[B76] Welte C. Deppenmeier U. (2011b). Re-evaluation of the function of the F_420_ dehydrogenase in electron transport of *Methanosarcina mazei*. FEBS J. 278, 1277–1287. 10.1111/j.1742-4658.2011.08048.x 21306561

[B77] Welte C. Deppenmeier U. (2014). Bioenergetics and anaerobic respiratory chains of aceticlastic methanogens. Biochim. Biophys. Acta 1837, 1130–1147. 10.1016/j.bbabio.2013.12.002 24333786

[B78] Yoshimatsu K. Araya O. Fujiwara T. (2007). *Haloarcula marismortui* cytochrome *b*-561 is encoded by the *narC* gene in the dissimilatory nitrate reductase operon. Extremophiles 11, 41–47. 10.1007/s00792-006-0016-3 16900298

[B79] Yoshimatsu K. Sakurai T. Fujiwara T. (2000). Purification and characterization of dissimilatory nitrate reductase from a denitrifying halophilic archaeon, *Haloarcula marismortui*. FEBS Lett. 470, 216–220. 10.1016/S0014-5793(00)01321-1 10734237

[B80] Zhu B. (2014). Microbial and Environmental Aspects of Anaerobic Oxidation of Methane. Ph.D. thesis, Radboud University.

