# Supplementary material for: A Metagenomics-Based Metabolic Model of Nitrate-Dependent Anaerobic Oxidation of Methane by Methanoperedens-Like Archaea
Source: Front Microbiol. 2015 Dec 18;6:1423. doi: 10.3389/fmicb.2015.01423 (PMC4683180; doi:10.3389/fmicb.2015.01423)
Supplement: Supplementary file 1 [file Table1.DOCX]

| **Taxonomy** | **Run 1**  **(read #)** | **Run 1**  **(%)** | **Run 2**  **(read #)** | **Run 2**  **(%)** | **Combined read (#)** | **Combined (%)** |
| --- | --- | --- | --- | --- | --- | --- |
| Methylomirabilis oxyfera | 576 | 30.7 | 1029 | 35.2 | 1605 | 33.4 |
| Methanoperedens nitroreducens | 403 | 21.5 | 649 | 22.2 | 1052 | 21.9 |
| Proteobacteria | 242 | 12.9 | 368 | 12.6 | 610 | 12.7 |
| Chloroflexi | 209 | 11.1 | 274 | 9.4 | 483 | 10.1 |
| Miscellaneous | 164 | 8.7 | 199 | 6.8 | 363 | 7.6 |
| Bacteroidetes | 121 | 6.4 | 138 | 4.7 | 259 | 5.4 |
| Acidobacteria | 71 | 3.8 | 132 | 4.5 | 203 | 4.2 |
| Planctomycetes | 38 | 2.0 | 92 | 3.1 | 130 | 2.7 |
| Armatimonadetes | 52 | 2.8 | 43 | 1.5 | 95 | 2.0 |

**Supplementary Table 1:** Analysis of the metagenome 16S rRNA gene read abundance. The two sequencing runs were analyzed separately (Run 1, Run 2) and the two analyses combined (Combined). The table lists read numbers (#) and relative amounts (per cent, %).
