# Supplementary material for: A Metagenomics-Based Metabolic Model of Nitrate-Dependent Anaerobic Oxidation of Methane by Methanoperedens-Like Archaea
Source: Front Microbiol. 2015 Dec 18;6:1423. doi: 10.3389/fmicb.2015.01423 (PMC4683180; doi:10.3389/fmicb.2015.01423)
Supplement: Supplementary file 2 [file Table2.DOCX]

| **Locus identifier** | **Protein subunit** | **Homolog in ANME_2D** | **% identity between ANME2D and MPEBLZ** | | **RPKM** | |
| --- | --- | --- | --- | --- | --- | --- |
| **F_420_H_2_dehydrogenase (Fpo)** | | | | | | |
| **MPEBLZ_00739**  **MPEBLZ_00738**  **MPEBLZ_00737**  **MPEBLZ_00736**  **MPEBLZ_00733**  **MPEBLZ_00732**  **MPEBLZ_00731**  **MPEBLZ_00730**  **MPEBLZ_00729**  **MPEBLZ_00728**  **MPEBLZ_00741**  **MPEBLZ_00742**  **MPEBLZ_00743**  **MPEBLZ_02422** | F_420_H_2_ dehydrogenase subunit FpoA  F_420_H_2_ dehydrogenase subunit FpoB  F_420_H_2_ dehydrogenase subunit FpoC  F_420_H_2_ dehydrogenase subunit FpoD  F_420_H_2_ dehydrogenase subunit FpoH  F_420_H_2_ dehydrogenase subunit FpoI  F_420_H_2_ dehydrogenase subunit FpoJ_2  F_420_H_2_ dehydrogenase subunit FpoJ_1  F_420_H_2_ dehydrogenase subunit FpoK  F_420_H_2_ dehydrogenase subunit FpoL  F_420_H_2_ dehydrogenase subunit FpoM  F_420_H_2_ dehydrogenase subunit FpoN  F_420_H_2_ dehydrogenase subunit FpoO  F_420_H_2_ dehydrogenase subunit FpoF | ANME2D_00974  ANME2D_00973  ANME2D_00972  ANME2D_00971  ANME2D_00970  ANME2D_00969  ANME2D_00968  ANME2D_00967  ANME2D_00966  ANME2D_00965  ANME2D_00964  ANME2D_00663  ANME2D_00662  ANME2D_02258 | 76  85  80  81  79  75  58  64  89  84  69  78  73  78 | 304  664  393  672  588  705  645  479  720  752  481  618  730  964 | |  |
| **Energy-conserving hydrogenase (Ech)** | | | | | | |
| **MPEBLZ_04052**  **MPEBLZ_04051**  **MPEBLZ_04043**  **MPEBLZ_04046**  **MPEBLZ_04044** | Energy-conserving hydrogenase subunit EchA  Energy-conserving hydrogenase subunit EchB  Energy-conserving hydrogenase subunit EchC  Energy-conserving hydrogenase subunit EchE  Energy-conserving hydrogenase subunit EchF | ANME2D_02724  ANME2D_02723  ANME2D_02718  ANME2D_02720  ANME2D_02719 | 63  78  92  78  71 | 52  58  65  80  69 | |  |
| **Cytoplasmic Heterodisulfide reductase (Hdr)** | | | | | | |
| **MPEBLZ_01151**  **MPEBLZ_01152**  **MPEBLZ_01153**  **MPEBLZ_01258**  **MPEBLZ_01259**  **MPEBLZ_01260**  **MPEBLZ_01179**  **MPEBLZ_01180**  **MPEBLZ_01181** | cytoplasmic heterodisulfide reductase subunit HdrC_1  cytoplasmic heterodisulfide reductase subunit HdrB_1  cytoplasmic heterodisulfide reductase subunit HdrA_1  cytoplasmic heterodisulfide reductase subunit HdrC_3  cytoplasmic heterodisulfide reductase subunit HdrB_3  cytoplasmic heterodisulfide reductase subunit HdrA_3  cytoplasmic heterodisulfide reductase subunit HdrC_2  cytoplasmic heterodisulfide reductase subunit HdrB_2  cytoplasmic heterodisulfide reductase subunit HdrA_2 | ANME2D_02156  ANME2D_02157  ANME2D_02158  ANME2D_02551  ANME2D_02552  ANME2D_02553  ANME2D_02156  ANME2D_02157  ANME2D_02158 | 80  81  84  71  81  83  81  80  85 | 369  313  335  482  227  137  152  283  237 | |  |
| **Membrane bound Hdr** |  |  |  |  | |  |
| **MPEBLZ_01018**  **MPEBLZ_01017** | membrane-bound heterodisulfide reductase subunit HdrD  membrane-bound heterodisulfide reductase subunit HdrE | ANME2D_02796  ANME2D_02797 | 83  67 | 946  1369 | |  |
| **F_420_reducing hydrogenase (Frh)** | |  |  |  | |  |
| **MPEBLZ_01158**  **MPEBLZ_02287** | F_420_-reducing hydrogenase subunit FrhB  F_420_-reducing hydrogenase subunit FrhB | ANME2D_02162  ANME2D_00417 | 86  30 | 368  217 | |  |
|  | |  |  |  | |  |
| **Methane oxidation to CO_2_ (reverse methanogenesis)** | |  |  |  | |  |
| **MPEBLZ_03394**  **MPEBLZ_01216**  **MPEBLZ_01217**  **MPEBLZ_01218**  **MPEBLZ_01219**  **MPEBLZ_01782**  **MPEBLZ_01173**  **MPEBLZ_04481**  **MPEBLZ_04482**  **MPEBLZ_02584**  **MPEBLZ_02585**  **MPEBLZ_02586**  **MPEBLZ_02587**  **MPEBLZ_01133**  **MPEBLZ_02588**  **MPEBLZ_02589**  **MPEBLZ_02590**  **MPEBLZ_02591**  **MPEBLZ_02677**  **MPEBLZ_02423**  **MPEBLZ_00120**  **MPEBLZ_01356**  **MPEBLZ_01201**  **MPEBLZ_01202**  **MPEBLZ_01203**  **MPEBLZ_01204**  **MPEBLZ_03729** | Formylmethanofuran--tetrahydromethanopterin formyltransferase  Molybdenum dependent formyl-MFR dehydrogenase subunit FmdC  Molybdenum dependent formyl-MFR dehydrogenase subunit FmdA  Molybdenum dependent formyl-MFR dehydrogenase subunit FmdB  Molybdenum dependent formyl-MFR dehydrogenase subunit FmdD  Molybdenum dependent formyl-MFR dehydrogenase subunit FmdE_2  Molybdenum dependent formyl-MFR dehydrogenase subunit FmdE_1  Tungsten dependent formyl-MFR dehydrogenase subunit FwdD  Tungsten dependent formyl-MFR dehydrogenase subunit FwdB  tetrahydromethanopterin S-methyltransferase subunit MtrH  tetrahydromethanopterin S-methyltransferase subunit MtrG  tetrahydromethanopterin S-methyltransferase subunit MtrF  tetrahydromethanopterin S-methyltransferase subunit MtrA  tetrahydromethanopterin S-methyltransferase subunit MtrA  tetrahydromethanopterin S-methyltransferase subunit MtrB  tetrahydromethanopterin S-methyltransferase subunit MtrC  tetrahydromethanopterin S-methyltransferase subunit MtrD  tetrahydromethanopterin S-methyltransferase subunit MtrE  Mtd: F_420_-dependent methylene H_4_MPT dehydrogenase  Mer: F_420_-dependent methylene –H_4_MPT reductase  Mch_1: Methenyl-H_4_MPT cyclohydrolase  Mch_2: Methenyl-H_~~4~~_MPT cyclohydrolase  Methyl-coenzyme M reductase subunit McrA  Methyl-coenzyme M reductase subunit McrG  Methyl-coenzyme M reductase subunit McrC  Methyl-coenzyme M reductase subunit McrB  Methyl-coenzyme M reductase protein C | ANME2D_00639  ANME2D_01681  ANME2D_01680  ANME2D_01679  ANME2D_01678  ANME2D_00408  ANME2D_00408  ANME2D_01940  ANME2D_01941  ANME2D_00495  ANME2D_00494  ANME2D_00493  ANME2D_00492  ANME2D_00492  ANME2D_00491  ANME2D_00490  ANME2D_00489  ANME2D_00488  ANME2D_01636  ANME2D_02259  ANME2D_02789  ANME2D_02789  ANME2D_01104  ANME2D_01103  ANME2D_01102  ANME2D_01101  ANME2D_00875 | 86  79  86  83  76  72  68  72  72  84  75  81  86  81  83  72  80  80  83  92  87  51  89  89  74  83  88 | 1333  691  404  511  296  18  446  319  162  1915  1505  2117  1201  369  1018  741  720  1268  1130  1575  374  160  57436  26015  45848  31898  1928 | |  |
| **Coenzyme biosynthesis** |  |  |  |  | |  |
| ***Coenzyme F_420_***  **MPEBLZ_03992**  **MPEBLZ_01508**  **MPEBLZ_03525**  **MPEBLZ_01413**  **MPEBLZ_01414**  ***Methanofuran***  **MPEBLZ_01550**  ***Methanopterin***  **MPEBLZ_03383**  **MPEBLZ_03533**  ***Coenzyme B***  **MPEBLZ_02136**  **MPEBLZ_00279**  **MPEBLZ_01066**  **MPEBLZ_03522**  **MPEBLZ_00997**  **MPEBLZ_01942**  **MPEBLZ_03757**  **MPEBLZ_01064**  **MPEBLZ_03706**  ***Coenzyme M***  **MPEBLZ_01988**  **MPEBLZ_00046**  **MPEBLZ_00708**  **MPEBLZ_03695**  **MPEBLZ_02613**  **MPEBLZ_01336** | CofE: conenzyme F420-0 gamma-glutamyl ligase  CofD: LPPG:Fo 2-phospho-_L_-lactate transferase  CofC: 2-phospho-L-lactate guanylyltransferase  CofG: Fo synthase subunit 1  CofH: Fo synthase subunit 2  CofA: Lactaldehyde dehydrogenase  CofB: _L_-Lactate kinase  MfnA: _L_-Tyrosine decarboxylase  MptA: GTP cyclohydrolase  MptB: Cyclic phosphodiesterase  Ribofuranosylaminobenzene 5’-P-synthase  leuA_2: Isopropylmalate synthase  leuA_1: Isopropylmalate synthase  leuB_1: Isopropylmalate dehydrogenase  leuB_2: Isopropylmalate dehydrogenase  LeuC_1: 3-isopropylmalate dehydratase, large subunit  LeuC_2: 3-isopropylmalate dehydratase, large subunit  LeuC_3: 3-isopropylmalate dehydratase, large subunit  LeuD1: 3-isopropylmalate dehydratase, small subunit  LeuD2: 3-isopropylmalate dehydratase, small subunit  thrC: Threonine synthase  thrC_2  thrC_3  thrC_5  aspartate aminotransferase  comDE: Sulfopyruvate decarboxylase | ANME2D_03361  ANME2D_02039  ANME2D_00233  ANME2D_00699  ANME2D_00698  ANME2D_01719  ANME2D_01328  ANME2D_03455  ANME2D_03335  ANME2D_01257  ANME2D_01547  ANME2D_01393  ANME2D_03321  ANME2D_03268  ANME2D_01549  ANME2D_01838  ANME2D_00244  ANME2D_00313  ANME2D_03037  ANME2D_00313  ANME2D_01156  ANME2D_03429 | 74  70  73  72  74  76  85  72  86  85  76  86  80  74  80  89  84  87  87  90  79  39  81  69 | 230  54  67  93  129  76  296  172  381  775  174  277  21  18  284  325  512  669  310  632  76  193  102  249 | |  |
| **Electron transport** |  |  |  |  | |  |
| **MPEBLZ_00485**  **MPEBLZ_01748**  **MPEBLZ_01947**  **MPEBLZ_03155**  **MPEBLZ_03192**  **MPEBLZ_03937**  **MPEBLZ_04240**  **MPEBLZ_00585**  **MPEBLZ_01373** | 4Fe-4S Ferredoxin  Ferredoxin  4Fe-4S Ferredoxin  4Fe-4S Ferredoxin  Ferredoxin  4Fe-4S Ferredoxin  4Fe-4S Ferredoxin  Ferredoxin  Polyferredoxin | ANME2D_03278  ANME2D_01372  ANME2D_03278  ANME2D_03050  ANME2D_03209  ANME2D_00885  ANME2D_03017  ANME2D_00764  ANME2D_01008 | 75  87  64  70  82  82  74  90  72 | 762  66  814  911  1643  98  835  14  78 | |  |
| **Acetate activation** |  |  |  |  | |  |
| **MPEBLZ_01317**  **MPEBLZ_01318**  **MPEBLZ_03841**  **MPEBLZ_03842**  **MPEBLZ_03843**  **MPEBLZ_03846**  **MPEBLZ_01103** | acetyl-CoA synthase subunit cdhD  acetyl-CoA synthase subunit cdhE  acetyl-CoA synthase subunit cdhA  acetyl-CoA synthase subunit cdhB  acetyl-CoA synthase subunit cdhC  acetyl-CoA synthase accessory protein CooC  acetyl-CoA synthetase | ANME2D_00521  ANME2D_00520  ANME2D_01197  ANME2D_01198  ANME2D_01199  ANME2D_01200  ANME2D_03105 | 90  86  88  79  84  82  79 | 620  554  482  202  541  93  152 | |  |
| **Quinone biosynthesis** |  |  |  |  | |  |
| **MPEBLZ_00322**  **MPEBLZ_02665**  **MPEBLZ_03084**  **MPEBLZ_02664**  **MPEBLZ_00825** | MqnA: chorismate dehydratase  MqnB: Futalosine hydrolase  MqnC: de-hypoxanthine futalosine cyclase  MqnD: 1,4-dihydroxy-6-naphtoate synthase  MqnE: aminofutalosine synthase | ANME2D_00530  ANME2D_02293  ANME2D_00949  ANME2D_02292  ANME2D_00937 | 77  61  76  69  78 | 102  125  117  100  91 | |  |
| **Oxidative phosphorylation: ATP synthase** | |  |  |  | |  |
| **MPEBLZ_01694**  **MPEBLZ_01695**  **MPEBLZ_01697**  **MPEBLZ_01698**  **MPEBLZ_01699**  **MPEBLZ_01700**  **MPEBLZ_01701**  **MPEBLZ_01702**  **MPEBLZ_01703** | (A1/A0)-ATP synthase subunit D  (A1/A0)-ATP synthase subunit B  (A1/A0)-ATP synthase subunit A  (A1/A0)-ATP synthase subunit F  (A1/A0)-ATP synthase subunit C  (A1/A0)-ATP synthase subunit E  (A1/A0)-ATP synthase subunit K  (A1/A0)-ATP synthase subunit I  (A1/A0)-ATP synthase subunit H | ANME2D_02066  ANME2D_02067  ANME2D_02068  ANME2D_02069  ANME2D_02070  ANME2D_02071  ANME2D_02072  ANME2D_02073  ANME2D_02074 | 89  94  88  77  78  67  76  72  60 | 1428  1034  933  494  1094  2136  4079  589  420 | |  |
| **Molybdenum cofactor biosynthesis** | |  |  |  | |  |
| **MPEBLZ_03870**  **MPEBLZ_03871**  **MPEBLZ_00500**  **MPEBLZ_02295**  **MPEBLZ_02297**  **MPEBLZ_02670**  **MPEBLZ_00045** | molybdopterin synthase subunit MoaD  Molybdopterin biosynthesis protein MoeA  bifunctional molybdopterin-guanine dinucleotide biosynthesis protein MoaE Molybdopterin-guanine dinucleotide biosynthesis protein A  MobB accessory protein  molybdopterin biosynthesis protein MoeA/LysR substrate binding-domain-containing protein  molybdenum cofactor biosynthesis protein MoaD | ANME2D_03403  ANME2D_03404  ANME2D_01900  ANME2D_01590  ANME2D_01588  ANME2D_02705  ANME2D_00314 | 60  73  72  64  73  72  88 | 69  59  103  122  22  56  1051 | |  |

**Supplementary Table 2:** Analysis of the proteome of *Methanoperedens nitroreducens* MPEBLZ in comparison to that of *M. nitroreducens* ANME2D. Presence and expression (RPKM value) of important enzymes in central metabolism and cofactor biosynthesis were analysed.
