# Supplementary material for: A Metagenomics-Based Metabolic Model of Nitrate-Dependent Anaerobic Oxidation of Methane by Methanoperedens-Like Archaea
Source: Front Microbiol. 2015 Dec 18;6:1423. doi: 10.3389/fmicb.2015.01423 (PMC4683180; doi:10.3389/fmicb.2015.01423)
Supplement: Supplementary file 3 [file Image1.PDF]

Supplementary Figure S1

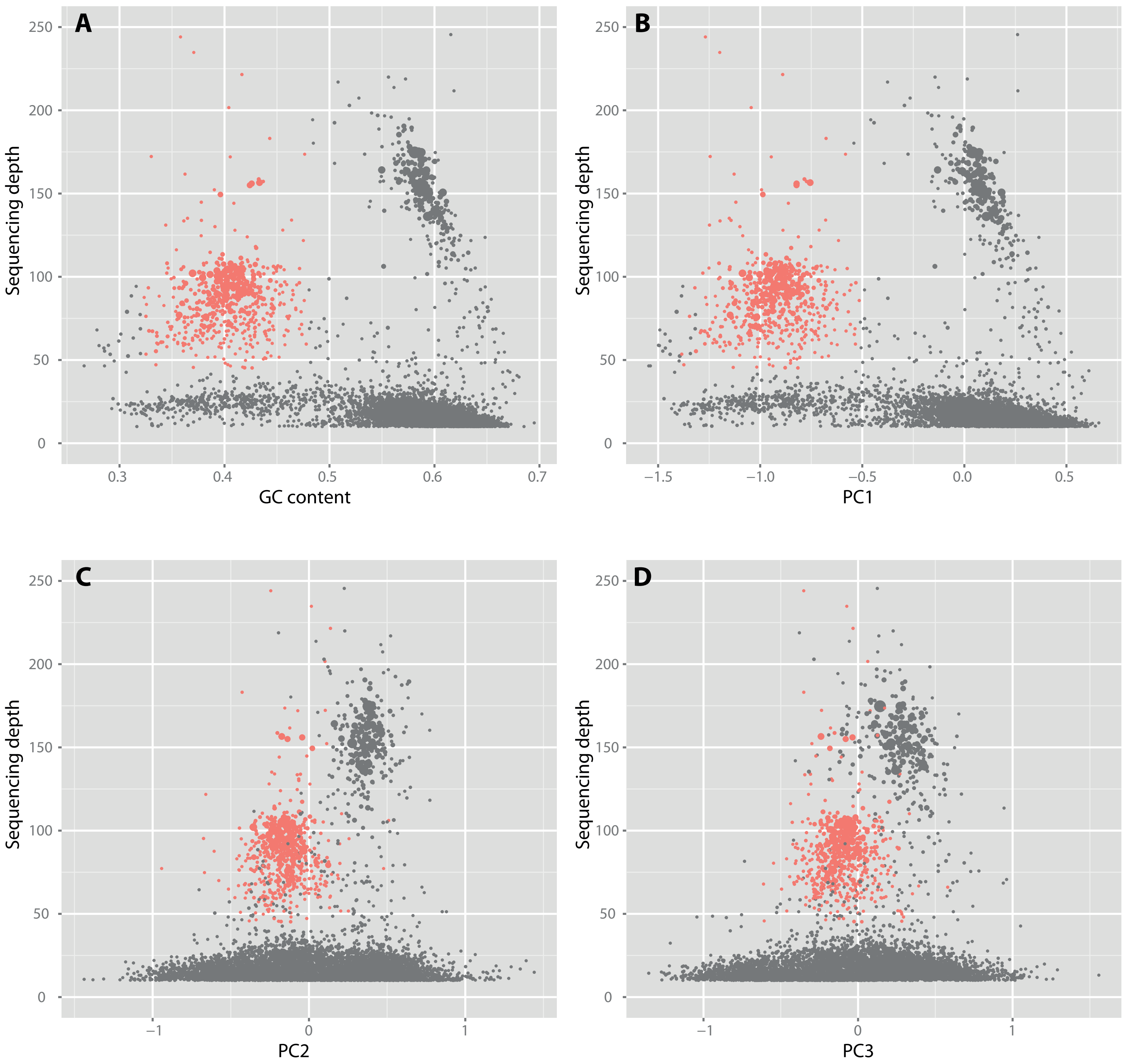

Supplementary Figure S1: Binning plots of the *Methanoperedens nitroreducens* BLZ1 draft genome

Scatterplots of the contigs with sequencing depth >10 assembled from the enrichment culture metagenome. Each point represents one contig, with size of the point proportional to contig length. The bin representing the Methanoperedens nitroreducens draft genome is indicated in red. Panel A shows the GC content of the contigs related to Sequencing depth, whereas panel B-D show the first 3 principle components of a PCA of tetranucleotide frequencies of the contigs in relation to the sequencing depth.
