# Supplementary material for: A Metagenomics-Based Metabolic Model of Nitrate-Dependent Anaerobic Oxidation of Methane by Methanoperedens-Like Archaea
Source: Front Microbiol. 2015 Dec 18;6:1423. doi: 10.3389/fmicb.2015.01423 (PMC4683180; doi:10.3389/fmicb.2015.01423)
Supplement: Supplementary file 4 [file Image2.PDF]

Supplementary Figure S2

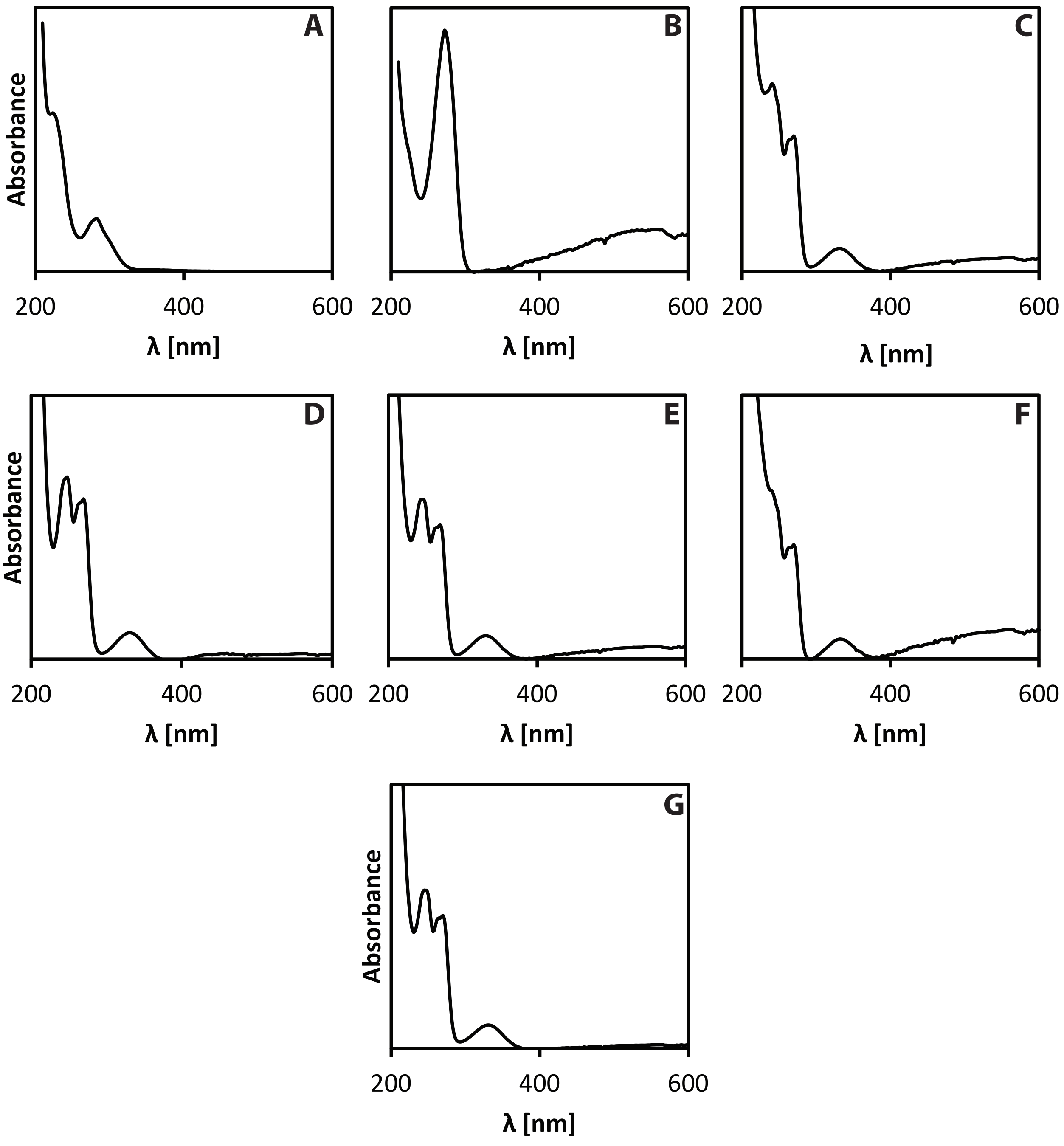

Supplementary Figure S2: Experimental UV/Vis spectra obtained from the fractions of the HPLC chromatogram described in Figure 3. All but one spectrum (spectrum B, peak 2) resemble the menaquinone standard spectrum (compare to Figure 2B). The images in (A) to (G) refer to the peak numbering 1 to 7 in Figure 3.
